# A Biomimetic C-Terminal Extension Strategy for Photocaging Amidated Neuropeptides

**DOI:** 10.1021/jacs.3c03913

**Published:** 2023-08-31

**Authors:** Aryanna E. Layden, Xiang Ma, Caroline A. Johnson, Xinyi J. He, Stanley A. Buczynski, Matthew R. Banghart

**Affiliations:** Department of Neurobiology, School of Biological Sciences, University of California San Diego, La Jolla, California 92093, United States

## Abstract

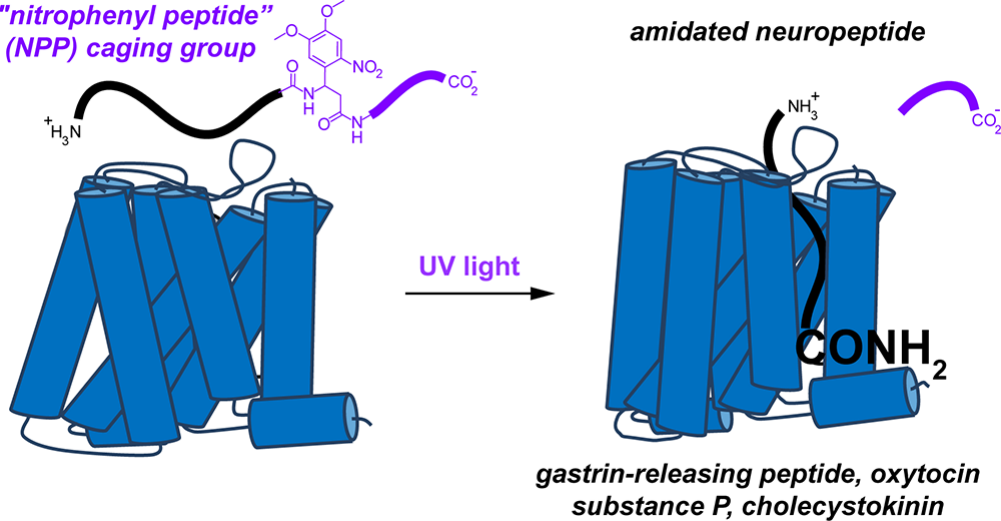

Photoactivatable neuropeptides offer a robust stimulus–response relationship that can drive mechanistic studies into the physiological mechanisms of neuropeptidergic transmission. The majority of neuropeptides contain a C-terminal amide, which offers a potentially general site for installation of a C-terminal caging group. Here, we report a biomimetic caging strategy in which the neuropeptide C-terminus is extended via a photocleavable amino acid to mimic the proneuropeptides found in large dense-core vesicles. We explored this approach with four prominent neuropeptides: gastrin-releasing peptide (GRP), oxytocin (OT), substance P (SP), and cholecystokinin (CCK). C-terminus extension greatly reduced the activity of all four peptides at heterologously expressed receptors. In cell type-specific electrophysiological recordings from acute brain slices, subsecond flashes of ultraviolet light produced rapidly activating membrane currents via activation of endogenous G protein-coupled receptors. Subsequent mechanistic studies with caged CCK revealed a role for extracellular proteases in shaping the temporal dynamics of CCK signaling, and a striking switch-like, cell-autonomous anti-opioid effect of transient CCK signaling in hippocampal parvalbumin interneurons. These results suggest that C-terminus extension with a photocleavable linker may be a general strategy for photocaging amidated neuropeptides and demonstrate how photocaged neuropeptides can provide mechanistic insights into neuropeptide signaling that are inaccessible using conventional approaches.

## Introduction

Neuropeptides comprise an abundant yet understudied class of neurotransmitter that activates G protein-coupled receptors (GPCRs) to modulate neuronal excitability, synaptic transmission, and neuroplasticity. Every neuron in the brain is likely capable of synthesizing and releasing one or more neuropeptides, in addition to a classical fast neurotransmitter such as glutamate, GABA, or acetylcholine.^1^ Indeed, peptides may have been used as neurotransmitters in primeval organisms before the evolution of complex nervous systems that contain synapses based on fast neurotransmission.^2^ Neuropeptides are derived from preproneuropeptide proteins that are synthesized at the soma and packaged into large dense-core vesicles (LDCVs) in the endoplasmic reticulum and Golgi apparatus. During LDCV trafficking and maturation, preproneuropeptides are proteolytically processed into active peptide fragments prior to Ca^2+^-dependent secretion. Historically, it has been difficult to faithfully stimulate and detect neuropeptide release to study peptidergic signaling. Most studies have therefore relied on bath application of peptide, which is too slow and spatially imprecise to be compatible with studies into the kinetics of receptor activation or peptide diffusion in neural tissue preparations. Bath application can also lead to widespread GPCR desensitization, which interferes with repeated measurements, thereby limiting experimental throughput.

To circumvent these limitations, we and others have developed several photoactivatable or “caged” neuropeptides. These include caged variants of the opioid peptides enkephalin and dynorphin,^3,4^ as well as somatostatin,^5^ orexin,^6^ and oxytocin.^7^ Caged molecules are advantageous because they can be pre-equilibrated in brain tissue in an inactive form prior to activation with millisecond flashes of light. Because pre-equilibration distributes the caged neuropeptide uniformly in the tissue, receptor activation by photoactivated peptide is not limited by diffusion. Accordingly, this photopharmacological approach can reveal receptor signaling kinetics at endogenous receptors in relatively intact tissue preparations such as brain slices. Because the photoreleased peptide is rapidly cleared by diffusion and proteolysis, receptor activation is transient and does not lead to extensive receptor desensitization, despite being able to fully saturate receptors. This feature enables experiments involving repeated peptide application over time, along with graded photoactivation to obtain a robust stimulus–response relationship that can be readily quantified and shaped as the basis of studies into signaling mechanisms.^8^ In addition, because light can be applied to tissue with exquisite spatial precision, caged neuropeptides are ideally suited for studies into volume transmission.^5^

Despite this utility, general strategies for generating caged neuropeptides are lacking. The most common strategy is to append a caging group to a single amino acid side chain that is deemed critical for receptor binding. However, suitable caging sites are unique to each peptide target and must be determined by the costly and laborious process of synthesizing and testing multiple analogues. Furthermore, some peptides lack cageable amino acid side chains that contribute strongly to receptor binding. In fact, fluorophore incorporation into some neuropeptide side chains can be well tolerated.^9−12^ Although, in principle, backbone amides can also be caged, the resulting sterically crowded molecules can be unstable and difficult to synthesize.^4,13^ Potentially general options include caging the N- or C-termini, depending on which end of the neuropeptide interacts most strongly with the receptor. Indeed, caging the N-terminal amine with a hydrolysis-resistant carbamate has proven effective for some peptides,^4^ yet in others, the N-terminus is solvent exposed such that caging is unlikely to reduce potency.

Most neuropeptides contain an amide at their C-terminus instead of a carboxylic acid. Neuropeptide amidation, which changes the charge of the C-terminus from negative to neutral, often contributes strongly to the biological activity of the peptide. Peptide amidation not only improves peptide stability but also influences molecular recognition at neuropeptide receptors.^14^ For example, replacement of the C-terminal amides in substance P, bombesin, peptide YY, cholecystokinin, and oxytocin with a carboxylic acid severely reduces receptor binding.^15−20^ This is consistent with the C-terminus of most amidated neuropeptides occupying space deep within the ligand-binding site, which has been implied for some time by biophysical studies^21^ and observed more recently in several ligand-bound receptor structures.^22−27^ Fortunately, unsubstituted amides can be readily generated from nitrobenzyl-derived caging groups that are stable in biological buffers.^28−30^ Thus, C-terminal amide caging is attractive as a potentially general caging strategy that could provide access to photoactivatable variants of more than half of known neuropeptides.

Here, we describe a biomimetic approach for producing light-activated amidated neuropeptides through caging of the C-terminal amide. Our strategy involves extending the C-termini of amidated neuropeptides with a photocleavable peptide such that photolysis mimics the final step of peptide biosynthesis. *In vitro* and *ex vivo* characterization of four C-terminally extended (C-TEx)-caged peptides suggests that the C-TEx-caging strategy is a highly generalizable approach for producing photocaged C-terminally amidated neuropeptides.

## Results and Discussion

### Design of a Biomimetic Caging Strategy

**Figure 1:**
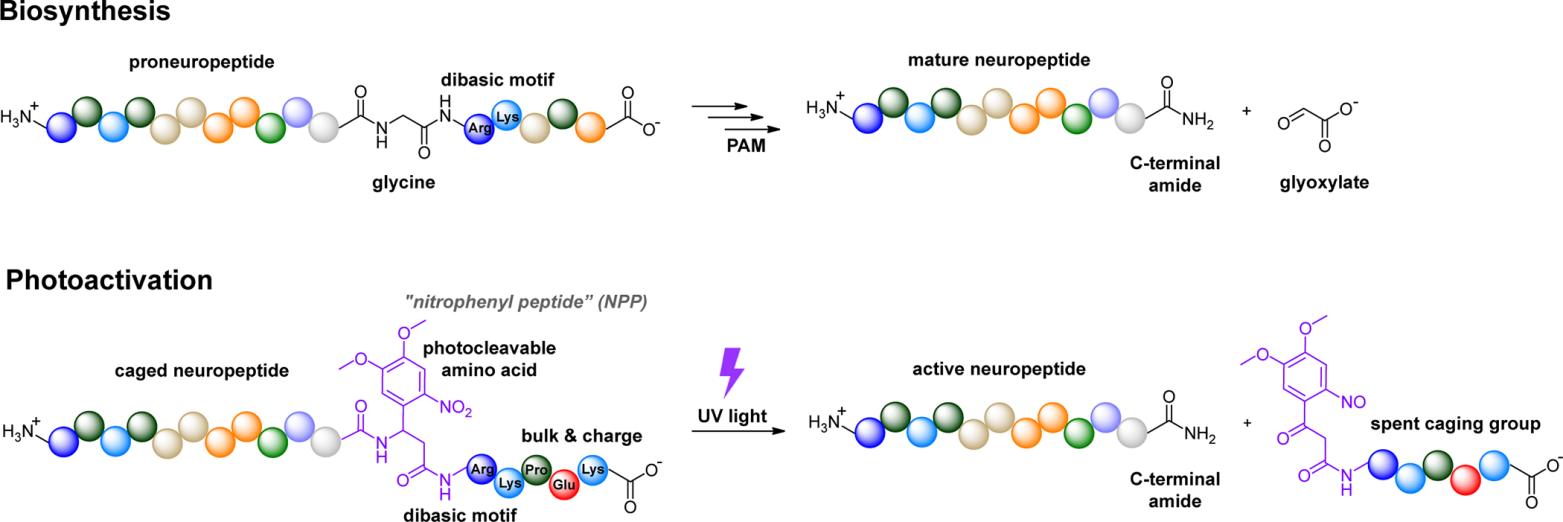
Biomimetic approach to photocaging C-terminally amidated neuropeptides. The biosynthesis of amidated neuropeptides involves enzymatic conversion of glycine to glyoxylate by peptidylglycine alpha-amidating monoxygenase (PAM). An adjacent dibasic motif initiates proteolytic processing prior to the oxidative cleavage by PAM to produce the active, C-terminally amidated neuropeptide. To mimic this process, the photocaged neuropeptide is C-terminally extended with a photocleavable amino acid followed by a dibasic motif and several charged, sterically bulky amino acids. Exposure to UV light removes the caging group to release the active, C-terminally amidated neuropeptide.

Our caging strategy was inspired by the biosynthesis of C-terminally amidated neuropeptides that occurs within large dense-core vesicles. C-terminus amidation is a posttranslational modification that involves an oxidative cleavage of a glycine residue to produce an unsubstituted amide (Figure 1). This transformation is carried out by an enzyme called peptidylglycine alpha-amidating monoxygenase (PAM) in the final step of C-terminus processing. Prior to activation by PAM, the proneuropeptide must be proteolytically processed at a pendent dibasic motif consisting of arginine and/or lysine residues that direct the activity of proteases such as prohormone convertases and carboxypeptidase E. Thus, proneuropeptides for C-terminally amidated neuropeptides contain a glycine followed by two basic amino acids immediately adjacent to the site of C-terminal amidation.

This motif is associated with immature neuropeptides that are likely not intended for receptor binding. Whether or not immature peptides are secreted from cells is not known, but we were encouraged by the finding that progastrin-releasing peptide does not bind to its receptors.^31^ We therefore reasoned that inclusion of the entire C-terminal amidation sequence might be particularly effective at reducing binding affinity if incorporated into C-terminally caged peptides, especially due to the presence of bulky, positively charged amino acids found in the dibasic motif. To mimic the proneuropeptide C-terminus, we substituted the glycine with dimethoxynitrobenzyl beta-alanine (DMNBA), a photocleavable amino acid, followed by an arginine-lysine dibasic motif. To accentuate the steric and electrostatic barriers to receptor binding, we added three additional amino acids: a proline to impart a sterically demanding kink, followed by glutamate and another lysine to impart additional bulk and charge. Collectively, we refer to this caging group as a “nitrophenyl peptide,” or NPP. Similar to the biosynthetic transformation mediated by proteases and PAM, illumination of an NPP-caged peptide leads to oxidation of the DMNBA beta-carbon to release a C-terminal amide, thus activating the synthetic peptide for receptor binding.

The DMNBA caging group is a dimethoxy-substituted variant of nitrobenzyl beta-alanine, an established photocleavable amino acid. We chose DMNBA due to its broad absorbance in the UV-A spectrum (315–410 nm) for which commercial LEDs are readily available, and because its utility in the context of peptide caging is less well established.

### Selection of Neuropeptide Targets

To explore general applicability of our biomimetic caging approach, we chose four prominent C-terminally amidated neuropeptides: gastrin-releasing peptide (GRP), oxytocin (OT), substance P (SP), and cholecystokinin (CCK). In the spinal cord, where it is released from sensory neurons, GRP is a potent mediator of itch.^32^ In the brain, GRP can function as a gatekeeper of cortical neuroplasticity through a disinhibitory mechanism.^33^ It is also involved in the regulation of food intake. OT plays important roles in childbirth and lactation, social bonding, and sexual function. It also has analgesic effects in the brain and spinal cord.^34,35^ Although several side chain-caged analogues of OT were reported recently,^7^ C-terminus caging has not been explored. SP contributes to the transmission of pain signals from the periphery to the central nervous system and to inflammation by promoting cytokine release. CCK stimulates digestion and suppresses food intake. In the context of pain modulation, it has a pronociceptive function through its anti-opioid actions.^36^

**Figure 2:**
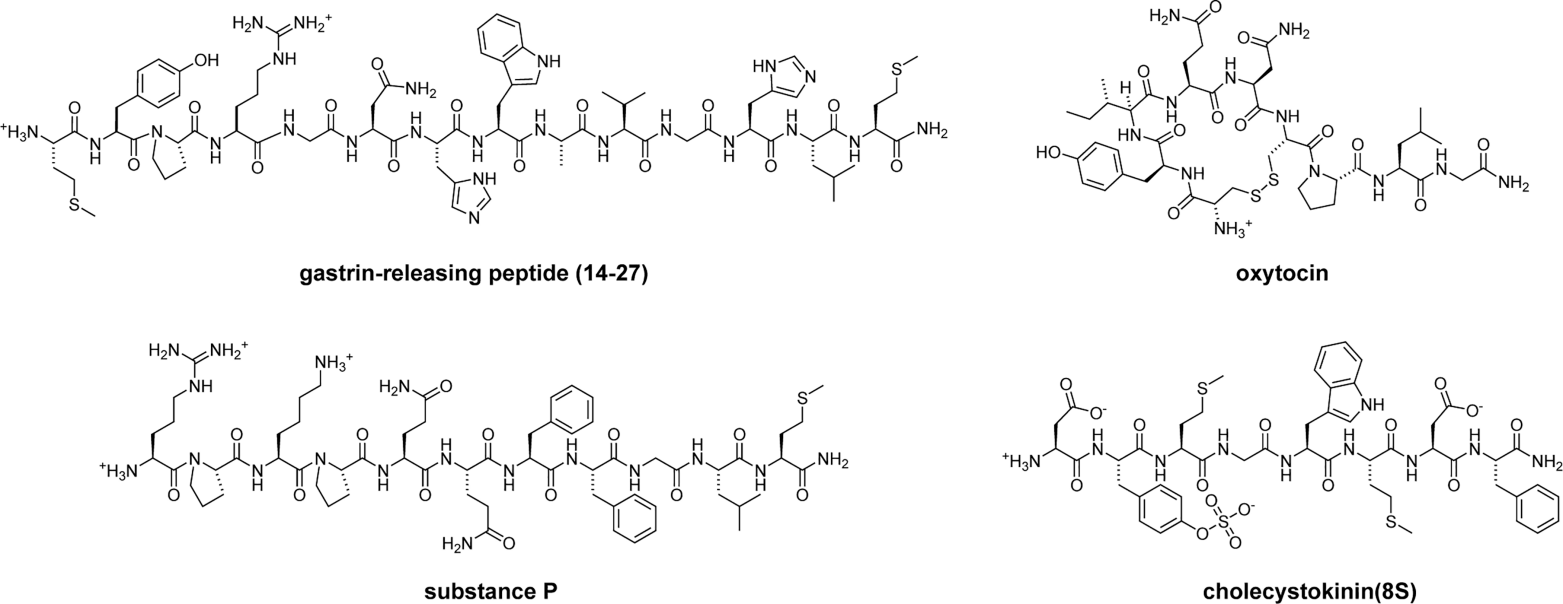
Chemical structures of gastrin releasing peptide (14-27), oxytocin, substance P, and cholecystokinin (8S). All four peptides contain a C-terminal amide but otherwise exhibit diverse structural features.

Each of these four peptides is a member of a different neuropeptide family. Other than the C-terminal amide, they lack sequence similarity and exhibit diverse chemical features (Figure 2). We chose to work with GRP(14-27), a naturally occurring C-terminal 14-amino acid fragment of full-length GRP that exhibits high potency and selectivity for the GRP receptor (GRPR). GRP(14–27) is a linear peptide that contains several polar amino acids and two positive charges, including the N-terminus. In contrast, OT is a cyclic peptide due to the presence of a disulfide bond between Cys1 and Cys6. It contains one positive charge on the N-terminus and several polar amino acids. Whereas the N-terminal half of SP, another linear peptide, is polar and positively charged, the C-terminal half, which interacts with the ligand binding site within the transmembrane domains of the neurokinin 1 receptor (NK1R), is hydrophobic and otherwise lacks cageable side chains. Several CCK variants that differ in length occur naturally. We chose to work with CCK(8S), the most abundant form in the brain. CCK(8S) contains a sulfated tyrosine residue near its C-terminus, along with two additional negatively charged amino acids.

### Synthesis and Chemical Characterization of NPP-Caged Neuropeptides

**Scheme 1:**
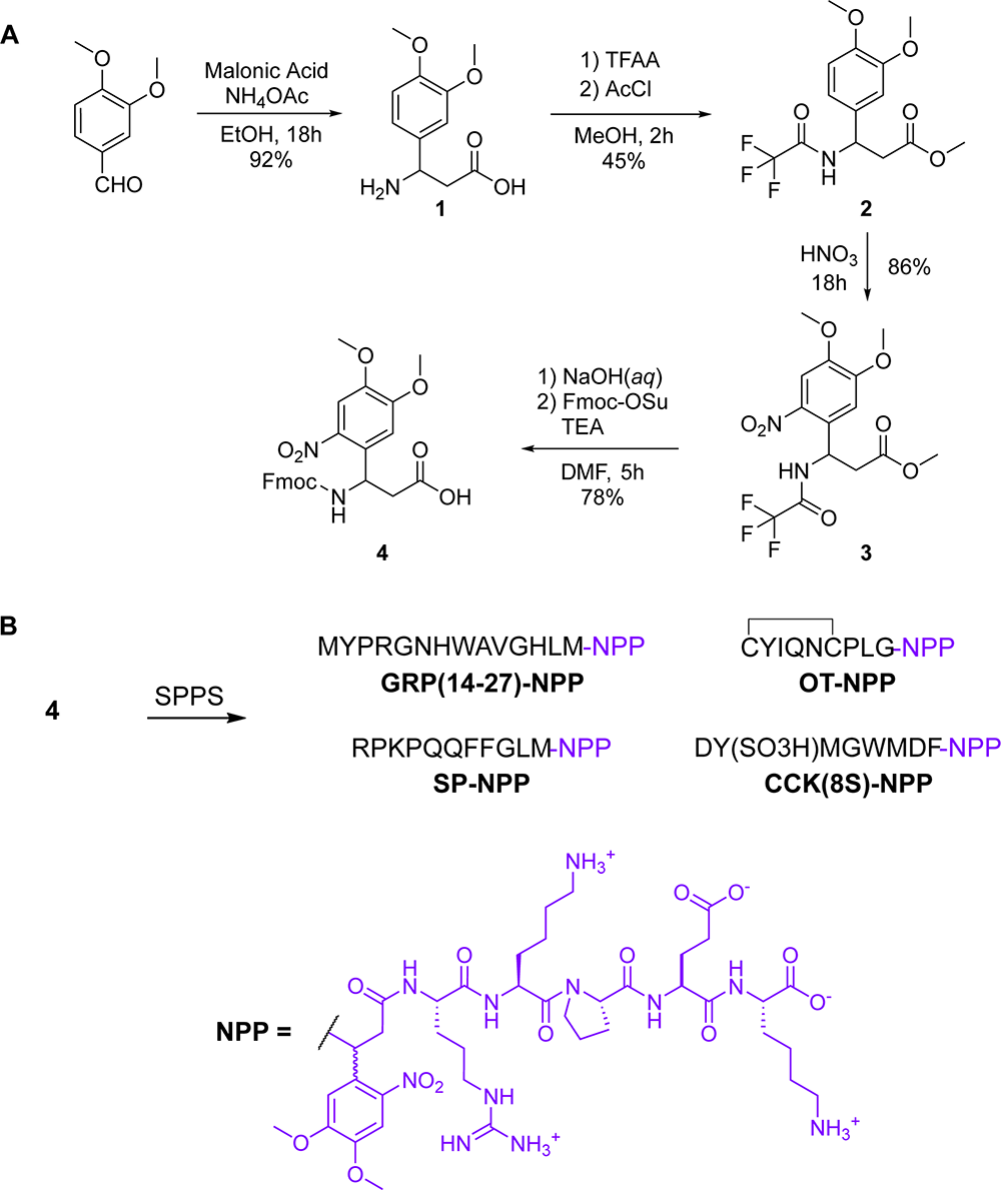
Synthesis of NPP-Caged Variants of Gastrin-Releasing Peptide (14-27), Oxytocin, Substance P, and Cholecystokinin (8S) (A) Fmoc-protected 2-(4,5-dimethoxy-2-nitrophenyl) beta-alanine (4) was prepared from 4,5-dimethoxybenzenaldehyde via a Mannich reaction followed by functional group protection and nitration. (B) NPP-caged peptides were prepared from 4 via solid phase peptide synthesis (SPPS).

Because we were unable to obtain good yields when attempting a Mannich reaction between 4,5-dimethoxy-2-nitrobenzaldehyde and malonic acid, we synthesized the DMNBA caging group using a slight modification to an existing protocol^37^ (Scheme 1A). Instead, Mannich reaction between malonic acid and 3,4-dimethoxybenzaldehyde proceeded smoothly under standard conditions to afford racemic 3-amino-3-(3,4-dimethoxyphenyl)propanoic acid (1) in 92% yield. We then devised a one-pot approach for protecting the amine and esterifying the carboxylic acid using trifluoroacetic anhydride, acetyl chloride, and methanol, which produced methyl 3-(3,4-dimethoxyphenyl)-3-(2,2,2-trifluoroacetamido)propanoate (2) in 45% yield. Subsequent nitration with nitric acid provided methyl 3-(4,5-dimethoxy-2-nitrophenyl)-3-(2,2,2-trifluoroacetamide)propanoate (3) in 86% yield. After removal of the trifluoroacetyl moiety and ester in aqueous NaOH, the free amine was Fmoc-protected to yield Fmoc-protected DMNBA (4), which was used to prepare the desired NPP-caged peptides via solid-phase peptide synthesis (Scheme 1B). In the cases of GRP(14-27)-NPP, OT-NPP, and CCK(8*S*)-NPP, diastereomers resulting from the use of racemic DMNBA were not resolved during purification. In contrast, the two diastereomers of SP-NPP (SP-NPP-ds1 and SP-NPP-ds2) were easily separated.

With the target NPP-caged peptides in hand, we examined their photochemical reactivity in cell-compatible aqueous solution (phosphate-buffered saline, pH 7.2). UV/vis spectroscopy revealed that all four NPP-caged peptides exhibited a peak absorbance wavelength ranging from 356 to 360 nm, which is typical for dimethoxynitrobenzyl caging groups (Supporting Figure 1A). HPLC analysis of samples taken during continuous illumination with 375 nm light showed that all four NPP-caged neuropeptides underwent photodegradation at similar rates (Supporting Figure 1B). Collectively, they were consumed 25–40% more slowly than an optical density-matched sample of MNI-glutamate, a commonly used UV-sensitive caged molecule that has a quantum yield of 0.065.^38^ LC–MS analysis verified that each NPP-caged peptide releases its corresponding parent peptide upon exposure to UV light (Supporting Figures 2–5). In addition to the desired neuropeptide products, with the exception of GRP(14-27)-NPP, we observed the formation of another peak that exhibited a shorter retention time than the NPP-caged peptides. We attribute this product to a side-reaction involving the DMNBA caging group (Supporting Figure 6). This side product retains the C-terminal extension and therefore likely exhibits similarly low biological activity to the NPP-caged peptides. Because the NPP-caged peptides released the intended neuropeptide products in roughly 50% chemical yield, we were encouraged to further examine their biological activity.

### *In Vitro* Analysis of NPP-Caged Neuropeptides

To determine the effectiveness of the C-TEx caging strategy at attenuating peptide activity, we conducted a live-cell functional assay of GPCR activation in HEK293T cells transfected to express the primary receptors for each neuropeptide. The GloSensor assay reports changes in cyclic adenosine monophosphate (cAMP) signaling as a function of GPCR activation. Because GRPR, OTR, NK1R, and CCK1R and CCK2R are all Gq-coupled GPCRs, their primary signal transduction mechanism does not directly involve changes in cAMP. To adapt this cAMP assay to report the activation of Gq-coupled GPCRs, we coexpressed a chimeric Gαs/Gαq protein (sq5) that allows Gq-coupled GPCRs to strongly engage the Gs pathway to elevate cAMP via activation of adenylyl cyclase.^39^

**Figure 3:**
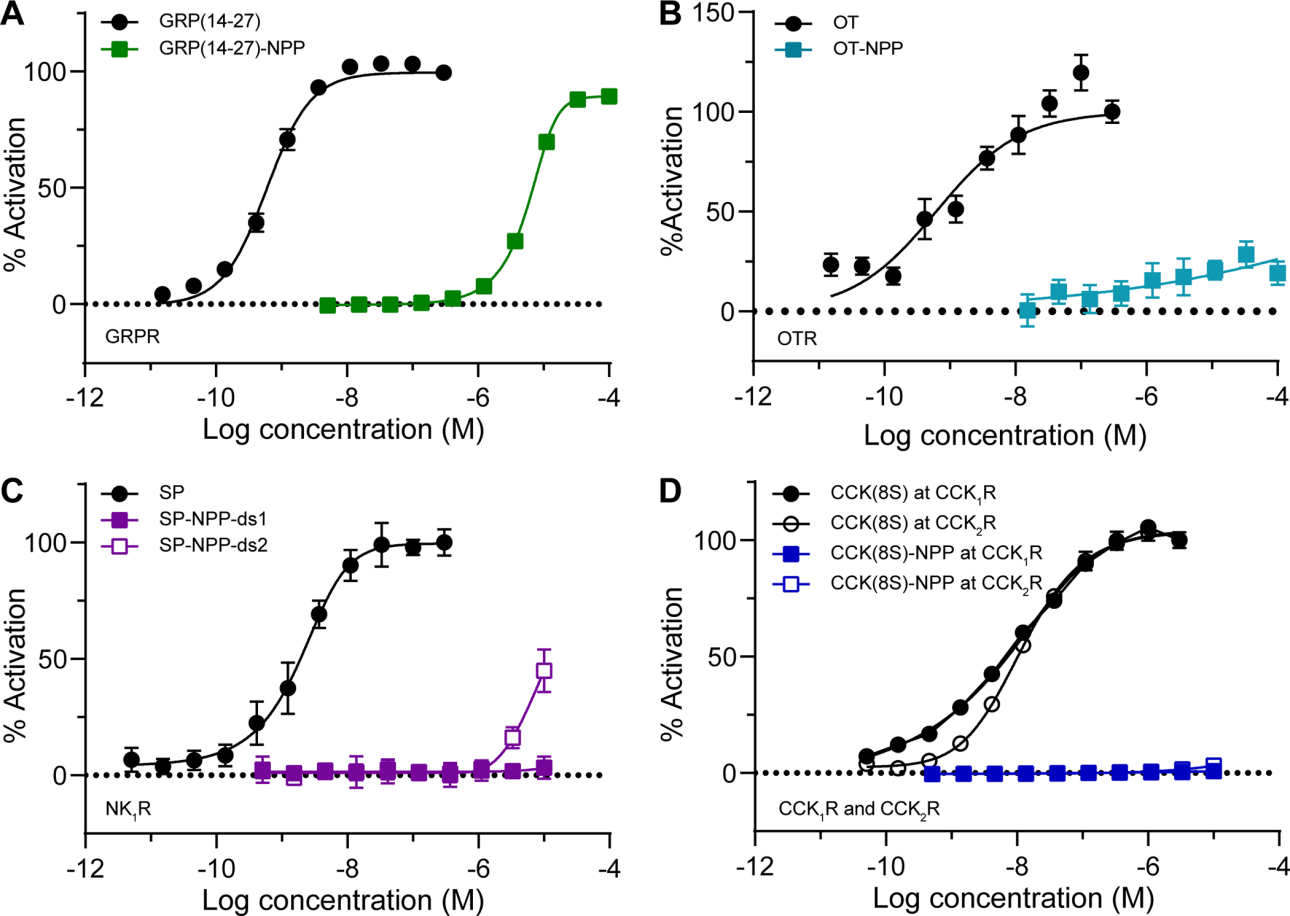
*In vitro* characterization of NPP-caged neuropeptides using a functional assay of G protein signaling. (A) Dose–response curves at the gastrin-releasing peptide receptor (GRPR) using a GloSensor assay of cAMP signaling in HEK293T cells (*n* = 10 wells per data point). Data were normalized to the maximal response to GRP(14-27) (300 nM) and are expressed as the mean ± SEM. (B) Same as panel A, but using the oxytocin receptor (OTR) and oxytocin (OT, 300 nM) for normalization. (C) Same as panel A, but using the neurokinin 1 receptor (NK1R) and substance P (SP, 300 nM) for normalization. (D) Same as panel A, but using the cholecystokinin receptors (CCK1R and CCK2R) and cholecystokinin-8S (CCK(8S), 300 nM) for normalization.

Dose–response curves were obtained for each NPP-caged peptide along with the unmodified parent peptide (Figure 3). In the case of GRP(14-27), NPP-caging reduced the EC50 at the GRPR by nearly 10,000-fold, from 0.60 nM to 6.1 μM. Although OT-NPP did not strongly activate the OTR at the highest concentrations tested, it exhibited weak but significant activity across the nM−μM concentration range, whereas OT exhibited an EC50 of 0.97 nM. Activation of the NK1R by both SP-NPP diastereomers was greatly reduced compared to SP (EC50 = 2.0 nM). Although SP-NPP-ds1 was devoid of activity at all concentrations tested, SP-NPP-ds2 produced ∼50% activation at the highest concentration tested (10 μM). We found CCK(8S) to activate CCK1R and CCK2R with similar affinities (CCK1R EC50 = 7.2 nM, CCK2R EC50 = 12 nM). In contrast, CCK(8*S*)-NPP did not detectably activate either receptor at concentrations up to 10 μM. Because SP-NPP-ds1 and CCK(8*S*)-NPP exhibited no agonism of their receptors, even at a concentration of 10 μM, we also determined that they do not antagonize their respective receptors (Supporting Figure 7). Based on these results, we conclude that the biomimetic C-TEx strategy is a viable, general approach to reducing the activity of C-terminally amidated neuropeptides.

### Photoactivation of Endogenous Neuropeptide Receptors in Brain Slices

Photoactivatable neuropeptides are powerful tools that can drive studies into the mechanisms of endogenous receptor signaling in acute brain slices.^3,8,40^ To determine if NPP-caged peptides are compatible with such experiments, we established electrophysiological assays for measuring the activation of GRPR, OTR, NK1R, and CCK2R in acute brain slices taken from transgenic mice. These assays involved whole cell recordings of membrane currents from genetically defined neurons located in different brain regions where endogenous receptor activation produces inward, excitatory currents that facilitate action potential firing. Neuropeptide receptor-expressing cells were identified for fluorescence-guided, targeted electrophysiological recordings using transgenic mice that contain transgenes encoding Cre-recombinase under a promotor that is specific to the cell of interest, as well as a Cre-dependent tdTomato fluorescent protein via the Ai14 (*Rosa26-lsl-tdTomato*) reporter strain.

Our experimental framework for evaluating NPP-caged peptides in brain slices is as follows. To assess residual activity, we compared the membrane currents evoked by bath application of the parent peptide and NPP-caged peptide. To evaluate photoactivation, we compared the peak current evoked by a single, high-intensity (20–200 ms, 50–80 mW) light flash from either a 355 nm laser or 365 nm LED, to the current evoked by bath application of the parent peptide, the NPP-caged peptide without light, and NPP-peptide photoactivation in the presence of a receptor antagonist.

**Figure 4:**
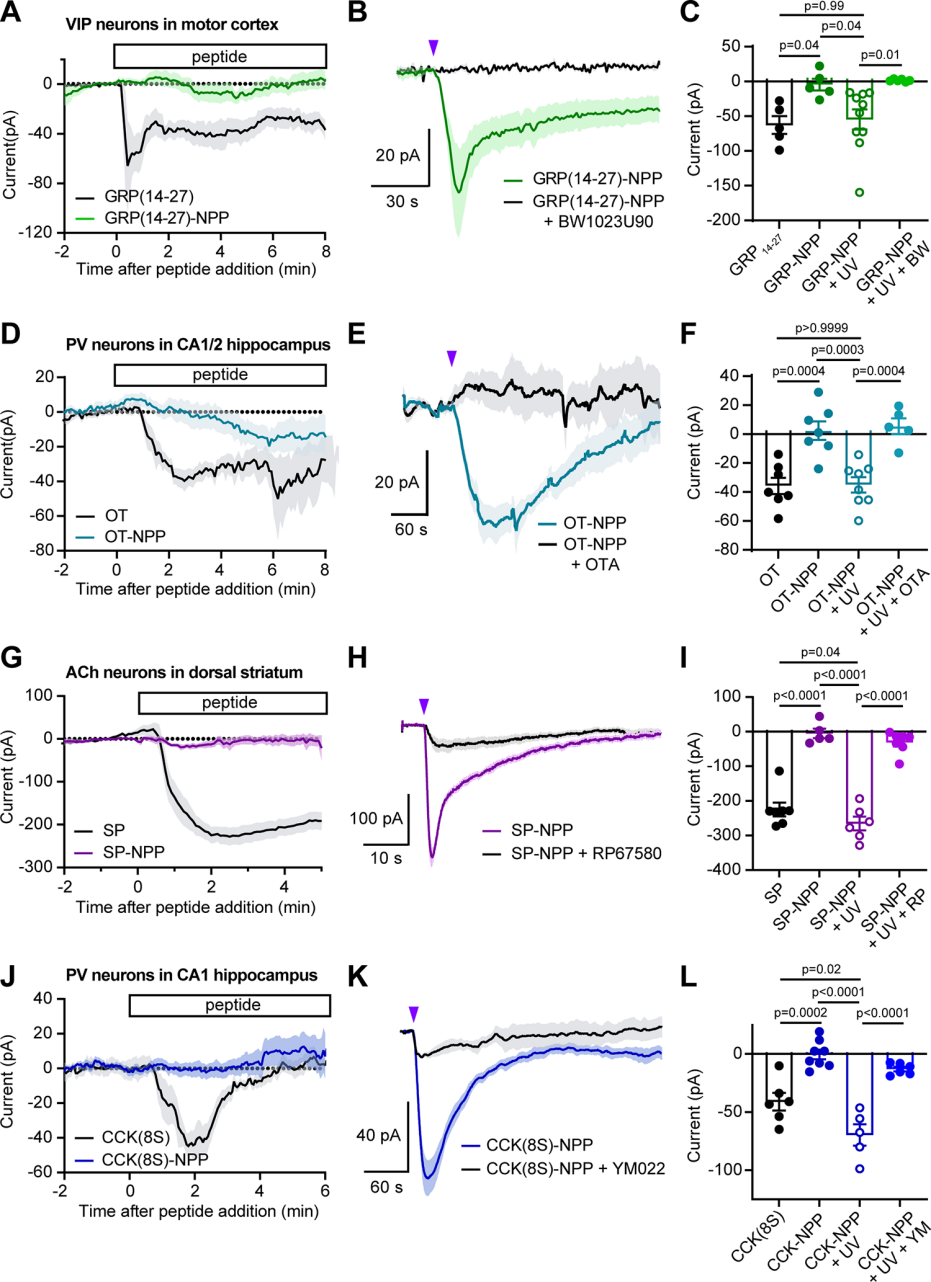
Electrophysiological validation of NPP-caged neuropeptides at endogenous receptors in acute brain slices. (A) Average inward currents over time after bath application of GRP(14-27)-NPP (3 μM, *n* = 5 cells from 4 mice) or GRP(14-27) (300 nM, *n* = 5 from 4 mice), recorded from fluorescently labeled VIP interneurons in layer 1 of the motor cortex. Data are expressed as the mean ± SEM. (B) Average inward currents evoked by photoactivation of GRP(14-27)-NPP (3 μM) with an 84 mW light flash in the absence (green, *n* = 10 cells from 4 mice) and presence of the GRPR antagonist BW1023U90 (1 μM) (black, *n* = 6 cells from 2 mice). (C) Summary of peak current amplitudes for the data shown in panels A and B. GRP(14-27) −62.8 ± 12.8; GRP(14-27)-NPP −4.8 ± 8.4; GRP(14-27)-NPP + UV −54.6 ± 14.4; GRP(14-27)-NPP + UV + BW1023U90 1.5 ± 0.7. Data are expressed as the mean ± SEM. Ordinary one-way ANOVA F (3, 22) = 6.37, *p* = 0.0028, *p*-values determined using Sidak’s multiple comparison’s test. (D) Average inward currents over time after bath application of OT-NPP (3 μM, *n* = 7 cells from 5 mice) or OT (300 nM, *n* = 7 from 4 mice), recorded from fluorescently labeled PV interneurons in the CA1 and CA2 regions of hippocampus. (E) Average inward currents evoked by photoactivation of OT-NPP (3 μM) with an 84 mW light flash in the absence (light blue, *n* = 8 cells from 4 mice) and presence of the OTR antagonist (OTA) (d(CH2)51,Tyr(Me)2,Thr4,Orn8,des-Gly-NH29)-Vasotocin (1 μM) (black, *n* = 5 cells from 2 mice). (F) Summary of peak current amplitudes for the data shown in panels D and E. OT −35.8 ± 5.7; OT-NPP 2.4 ± 6.5; OT-NPP + UV −35.1 ± 5.3; OT-NPP + UV + OTA 5.5 ± 5.5. Data are expressed as the mean ± SEM. Ordinary one-way ANOVA F (3, 23) = 14.83, *p* <0.0001, *p*-values determined using Sidak’s multiple comparison’s test. (G) Average inward currents over time after bath application of SP-NPP (1 μM, *n* = 6 cells from 5 mice) or SP (500 nM, *n* = 7 from 2 mice), recorded from fluorescently labeled cholinergic interneurons in the dorsal striatum. (H) Average inward currents evoked by photoactivation of SP-NPP (1 μM) with an 84 mW light flash in the absence (purple, *n* = 5 cells from 5 mice) and presence of the NK1R antagonist RP67580 (10 μM) (black, *n* = 6 cells from 3 mice). (I) Summary of peak current amplitudes for the data shown in panels G and H. SP −197.8 ± 20.1; SP-NPP −5.2 ± 13.6; SP-NPP + UV −265.5 ± 19.7; SP-NPP + UV + RP67580 −30. 9 ± 11.7. Data are expressed as the mean ± SEM. Ordinary one-way ANOVA F (3, 21) = 53.13, *p* <0.0001, *p*-values determined using Sidak’s multiple comparison’s test. (J) Average inward currents over time after bath application of CCK(8*S*)-NPP (3 μM, *n* = 8 cells from 6 mice) or CCK(8S) (500 nM, *n* = 6 from 3 mice), recorded from fluorescently labeled PV interneurons in the CA1 region of hippocampus. (K) Average inward currents evoked by photoactivation of CCK(8*S*)-NPP (3 μM) with an 84 mW light flash in the absence (blue, *n* = 5 cells from 3 mice) and presence of the CCK2R antagonist YM022 (1 μM) (black, *n* = 6 cells from 2 mice). (L) Summary of peak current amplitudes for the data shown in panels J and K. CCK −41.1 ± 7.6; CCK-NPP −0.4 ± 4.1; CCK-NPP + UV −69.6 ± 9.2; CCK-NPP + UV + 1 uM YM 022 −13.0 ± 2.0. Data are expressed as the mean ± SEM. Ordinary one-way ANOVA F (3, 21) = 26.59, *p* <0.0001, *p*-values determined using Sidak’s multiple comparison’s test.

To evaluate GRP(14-27)-NPP, we recorded from vasoactive intestinal peptide (VIP)-expressing neurons in primary motor cortex, which express GRPR,^33^ in brain slices taken from *Vip^Cre^*/*Rosa26-lsl-tdTomato* mice (Figure 4A–C). Whereas GRP(14-27) (300 nM) produced an inward current that desensitized over the course of several minutes (Figure 4A), GRP(14-27)-NPP (3 μM) did not produce a significant response. Application of a single, high-intensity (1 × 20 ms, 84 mW, 355 nm laser) light flash evoked a rapidly activating current (τ_on_ = 10.1 s) that deactivated over the course of several minutes with biphasic kinetics (τ_off_^fast^ = 3.9 s, τ_off_^slow^ = 44.6 s). The peak amplitude of the light-evoked current was similar in amplitude to that produced by GRP(14-27) bath application (1 μM). Consistent with the current resulting from activation of GRPR, the response was completely blocked by the GRPR antagonist BW1023U90 (1 μM) (Figure 4B). These results are summarized in Figure 4C.

We evaluated OT-NPP by recording from parvalbumin (PV)-expressing neurons in the CA1 and CA2 regions of hippocampus, which express OTR, in brain slices taken from *Pvalb^Cre^*/*Rosa26-lsl-tdTomato* mice^41^ (Figure 4D–F). Although OT (300 nM) produced an inward current within 3–4 min of addition to the bath, OT-NPP (3 μM) did not (Figure 4D). However, consistent with the partial residual activity observed *in vitro*, a small current was detected 6–8 min later. Application of a UV light flash (1 × 20 ms, 84 mW, 355 nm laser) evoked a current that activated and deactivated with surprisingly slow kinetics (τ_on_ = 46.6 s, τ_off_ = 235.5 s) and was blocked by the OTR antagonist (OTA) (d(CH2)_5_^1^,Tyr(Me)^2^,Thr^4^,Orn^8^,des-Gly-NH_2_^9^)-Vasotocin (1 μM) (Figure 4E, summarized in Figure 4F).

We evaluated SP-NPP (1:1 mixture of diastereomers) by recording from striatal cholinergic interneurons (CINs), which express NK1R,^42^ in brain slices taken from *Chat^Cre^/Rosa26-lsl-tdTomato* mice (Figure 4G–I). Whereas SP (500 nM) produced a large, sustained inward current, SP-NPP (3 μM) was inactive (Figure 4G). Photoactivation (1 × 20 ms, 84 mW, 365 nm LED) generated a large, rapidly activating current that exhibited biphasic decay kinetics (τ_on_ = 1.1 s, τ_off_^fast^ = 1.4 s, τ_off_^slow^ = 19.5 s). The light-evoked response was largely blocked by the NK1R antagonist RP67580 (10 μM) (Figure 4H, summarized in Figure 4I).

To evaluate CCK(8*S*)-NPP, we again recorded from PV-expressing neurons, which express CCK2R, in the CA1 region of hippocampus^43^ (Figure 4J–L). Whereas bath application of CCK(8S) (500 nM) produced a small inward current that fully desensitized within several minutes of activation, CCK(8*S*)-NPP (3 μM) was inactive (Figure 4J). Photoactivation (1 × 200 ms, 84 mW, 355 nm laser) evoked a current that activated and inactivated with rapid kinetics (τ_on_ = 16.0 s, τ_off_ = 41.7 s) and was strongly attenuated by the CCK2R antagonist YM022 (Figure 4K). Surprisingly, the light-evoked current was larger in amplitude than the current evoked by a saturating concentration of CCK(8S) (Figure 4L). This is likely due to the rapid accumulation of receptor desensitization during the relatively slow phase of diffusion-limited receptor activation that occurs with bath application of peptide. In contrast, the rapid concentration jump achieved by photoactivation reveals the maximal response that is not attenuated by simultaneous desensitization.

### CCK(8*S*)-NPP Reveals Mechanisms of CCK Signaling in Hippocampal PV Interneurons

Encouraged by these results, we used CCK(8*S*)-NPP to probe two poorly understood aspects of CCK signaling: the role of peptidases in limiting peptidergic transmission, and functional interactions between CCK receptors and opioid receptors. These topics are difficult to address using standard peptide application methods due to the modest size of most peptide-driven membrane currents coupled with slow diffusion of experimentalist-applied peptides in and out of brain tissue. Changes in small, slow, peptide-evoked currents (10’s of pA over minutes) are hard to reliably quantify due to intrinsic drift in holding currents on the same scales during electrophysiological recordings. Rapid agonist application with light minimizes the time of such experiments such that only short baselines are required, which minimizes confounds due to drift. Furthermore, the highly stereotyped pulse of agonist that results from photorelease produces a robust response profile with many features that can be quantified to detect changes in signaling: amplitude, activation and deactivation kinetics, and integrated response over a defined time window.

Although extracellular peptidases are known to limit peptide concentrations in the nervous system, how they shape the temporal dynamics of neuropeptide signaling in the brain is poorly understood. For example, rapid proteolysis by peptidases localized near peptide receptors might limit the peak concentration of peptide and thus the degree of receptor activation. In *locus coeruleus*, peptidase inhibition increases the potency of bath applied enkephalin 10-fold,^44^ but only potentiates enkephalin photouncaging responses to volumetrically large stimuli.^3^ These observations suggest that diffusion rather than proteolysis is the primary mechanism of enkephalin clearance in the *locus coeruleus*, but how peptidases contol neuropeptide signaling in other contexts requires further investigation.

**Figure 5:**
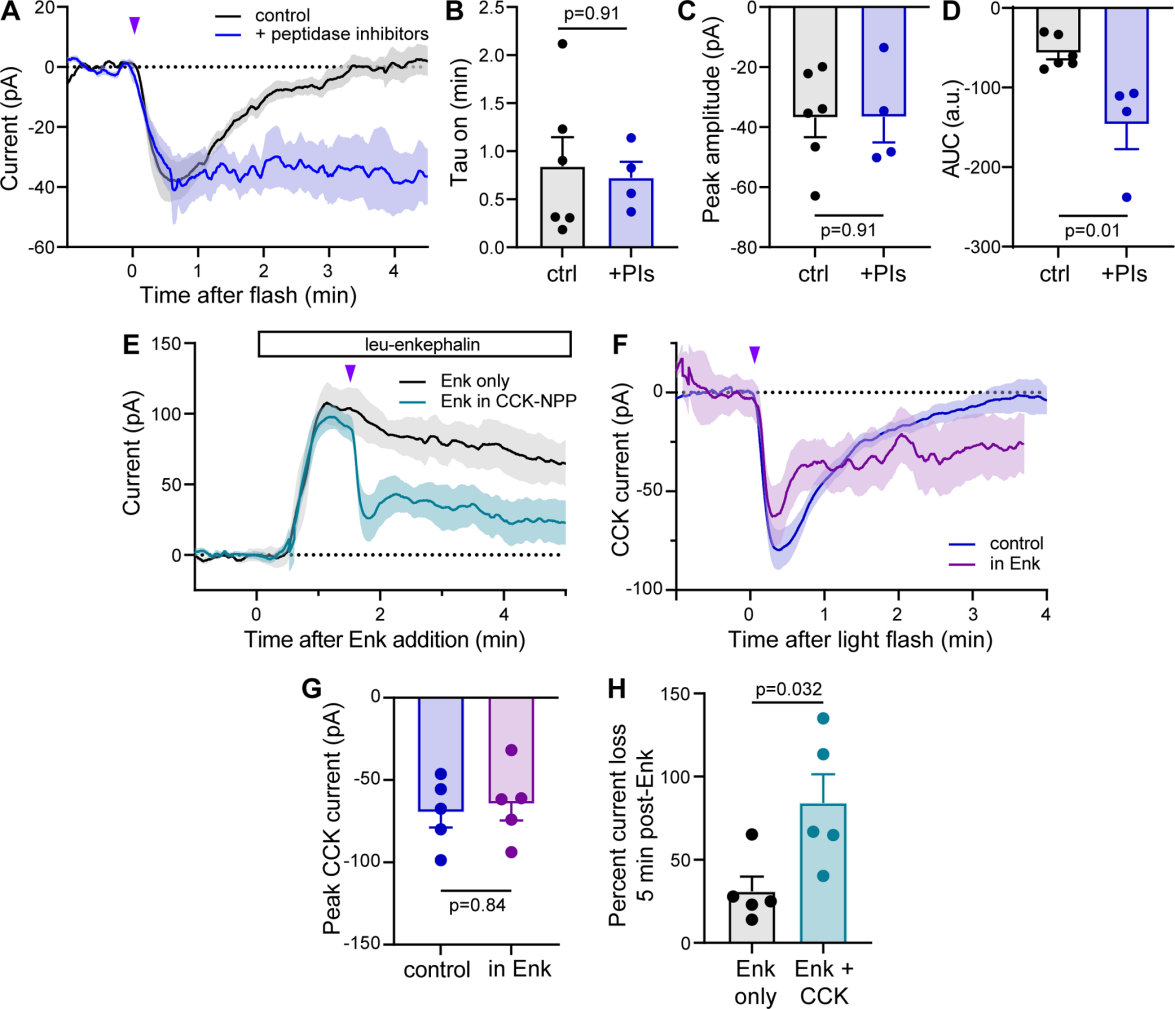
CCK(8*S*)-NPP photouncaging unmasks temporal features of CCK signaling. (A) Average inward currents evoked by photoactivation of CCK(8*S*)-NPP (3 μM) with a 20 ms, 84 mW light flash in the absence (black, *n* = 6 cells from 2 mice) and presence of a cocktail of peptidase inhibitors (phosphoramidon (1 μM), bestatin (20 μM), butabindide ((2 μM)) (blue, *n* = 4 cells from 2 mice), recorded from fluorescently labeled PV interneurons in the CA1 region of hippocampus. Data are expressed as the mean ± SEM. (B) Summary of current activation time constants for the data shown in panel A. control 0.84 ± 0.30; PIs 0.73 ± 0.17. Data are expressed as the mean ± SEM. Mann–Whitney test. (C) Summary of peak current amplitudes for the data shown in panel A. control −36.8 ± 16.0; PIs −36.6 ± 16.9. Data are expressed as the mean ± SEM. Mann–Whitney test. (D) Summary of the area under the curve (0–4.5 min postflash) for the data shown in panel A. control −56.6 ± 19.9; PIs −146.5 ± 61.7. Data are expressed as the mean ± SEM. Mann–Whitney test. (E) Average outward currents evoked by bath application of leucine-enkephalin (Enk, 1 μM) in the absence (black, *n* = 5 cells from 2 mice) and presence of subsequent CCK(8*S*)-NPP (3 μM) uncaging (1 × 200 ms, 84 mW flash, teal, n = 5 cells from two mice). (F) Average inward current evoked by photoactivation of CCK(8*S*)-NPP in the absence (blue (control), same data as Figure 4K) and presence of Enk (purple, *n* = 5 cells from 2 mice), subtractively isolated from the data shown in panel E. (G) Summary of peak current amplitudes for the data shown in panel F. Control −69.6 ± 20.6; in Enk −64.5 ± 22.6. Data are expressed as the mean ± SEM. Mann–Whitney test. (H) Summary of percent current loss 5 min after Enk addition for the data shown in panel F. Enk only 31.0 ± 19.8; Enk + CCK photorelease 84.1 ± 38.8. Data are expressed as the mean ± SEM. Mann–Whitney test.

To determine how peptidases impact CCK signaling in hippocampus, we photoactivated CCK(8*S*)-NPP with a modest, subsaturating optical stimulus (1 × 20 ms, 84 mW, rather than 1 × 200 ms, 84 mW), in the absence and presence of a cocktail of drugs that inhibit peptidases known to degrade CCK.^45−47^ Strikingly, inclusion of the peptidase inhibitors (PIs) dramatically reduced the deactivation kinetics of the CCK(8*S*)-induced current, without impacting either the activation kinetics or the amplitude of the photouncaging response (Figure 5A–C). Because peptidase inhibition produced a step-like response to CCK(8S) uncaging that did not decay for at least 4 min after the light flash, a deactivation time-constant could not be determined. Instead, this temporal potentiation was captured in the response integral (area under the curve, Figure 5D). These results reveal that peptidases define the duration, but not the magnitude of CCK(8S) signaling in hippocampal PV interneurons.

CCK has long been considered an “anti-opioid” neuropeptide due to its suppression of opioid antinociception.^36,48,49^ Although antagonistic CCK-opioid interactions can occur at the circuit level,^50^ the cellular and molecular mechanisms of their cell-autonomous functional interactions remain poorly understood. CCK(8S) has been reported to reduce agonist binding to opioid receptors and agonist-induced intracellular signal transduction,^51^ both of which could be accounted for by heteromerization of CCK- and opioid receptors.^52^ In addition to CCK2Rs, hippocampal PV interneurons express mu and delta opioid receptors (MOR and DOR), the activation of which produces outward membrane currents mediated by G protein-coupled inward rectifier K^+^ (GIRK) channels.^8^ In contrast, the inward current produced by CCK2R activation in PV interneurons is mediated by Ca^2+^-activated Na^+^-conducting transient receptor potential (TRP) channels.^43^

To explore potential functional interactions between CCK- and opioid receptor signaling in PV interneurons, we photoactivated CCK(8*S*)-NPP using a strong optical stimulus (1 × 200 ms, 84 mW flash) 1.5 min after bath application of [Leu]^5^-enkephalin (Enk, 1 μM), which activates both MOR and DOR (Figure 5E). As expected, CCK(8S) photorelease drove a large, rapid reduction in the Enk-evoked GIRK current. Because the opioid current desensitizes over the course of several minutes, we isolated the average CCK(8S) component by subtracting away the average current produced by Enk alone. Surprisingly, comparison to the CCK(8S) uncaging response in control conditions revealed an apparent potentiation of the CCK(8S) response by concomitant opioid receptor signaling (Figure 5F). Similar to the action of peptidases, Enk appeared to prolong the CCK(8*S*)-evoked current without altering the amplitude of the uncaging response (Figure 5G). Viewed from the perspective of CCK2R signaling, this finding seems to contradict the notion of antagonistic CCK and opioid receptor signaling. However, because the apparent CCK(8S)-current is isolated from a background opioid current, an alternative interpretation is that short-lived CCK2R signaling drove a sustained inhibition of the opioid-mediated GIRK current. In control conditions, the CCK(8*S*)-evoked current returns to baseline ∼3 min after the light flash. In the absence of functional interactions, the Enk-evoked outward current (Figure 5E, teal line) would thus be expected to recover from CCK2R signaling and merge with the Enk control current (Figure 5E, black line) ∼4.5 min after Enk addition. Instead, the opioid current was suppressed to a greater degree than that produced by receptor desensitization alone (Figure 5H). These findings are consistent with CCK signaling exhibiting a “switch-like” anti-opioid effect, wherein transient CCK2R activation triggers long-lasting deactivation of opioid receptor signaling.

## Conclusions

Together, these findings demonstrate that the C-TEx strategy is a viable and broadly applicable approach to photocaging structurally diverse amidated neuropeptides. Furthermore, our results validate four new photopharmacological tools that can be used to probe neuropeptide signaling with high spatiotemporal precision. Our biomimetic design is based on the biosynthesis of native amidated neuropeptides. A recent study found that addition of a single caging group to the C-terminal amide of orexin sufficiently inactivated the peptide at the Orexin B receptor for subsequent photouncaging.^6^ Although small C-terminal caging groups may suffice for some peptide targets, it is not currently clear how well this minimal perturbation will generalize to other peptides and receptors. Our findings suggest that C-terminal extension with a large, charged, and sterically bulky peptide offers a reliable yet general strategy for quickly generating useful reagents for multiple peptide signaling pathways.

While our study establishes the feasibility of our approach, future work is required to optimize the amide caging chemistry. Using the DMNPA caging group, we observed the formation of a side-product that reduces the chemical yield of the photoreleased peptide. The proposed byproduct appears to result from the presence of the 4-methoxy group on DMNPA, which suggests that implementation of alternative caging groups should improve the light sensitivity of C-TEx-caged peptides. Of note, amide-compatible alternatives to the nitrobenzyl family, including caging groups that respond to longer wavelengths of light, remain to be established. In addition, because NPP-caged neuropeptides are not protected against proteolysis, which may lead to degradation prior to photoactivation, they may find limited use *in vivo*, where the quantity of delivered peptide is limiting. Identifying caging strategies that simultaneously prevent GPCR binding and protease activity should thus be an important future goal.

Recent progress encapsulating neuropeptides in light-sensitive nanovesicles offers a potentially general approach to caging peptides with even less reliance on chemical structure.^5^ Encapsulation also protects caged peptides from extracellular proteases. However, current nanovesicle formulations do not distribute well in brain tissue, which limits sites of photorelease to those at which vesicles happen to be deposited. Furthermore, nanovesicle contents are depleted by repeated photoactivation. In contrast, soluble-caged peptides distribute uniformly in tissue, especially in brain slices that are bathed in a large excess of reagent. After photoactivation within an illuminated volume, fresh caged peptide replaces the consumed reagent through diffusion, such that the response to repeated illumination is highly reproducible within a single experiment.^3,8^ Each approach offers unique strengths and weaknesses that may dictate their use in different contexts.

Although caged neurotransmitters have played an important role in neuroscience research for decades, caged neuropeptides have only recently been used to drive studies into peptide biology.^8^ As demonstrated here in the contexts of peptide proteolysis and receptor crosstalk, experimental shaping of the well-defined stimulus–response relationship provided by peptide photoactivation can yield mechanistic insights that would be otherwise obscured by the slow kinetics of peptide diffusion. In addition, caged peptides are well-suited for studies into volume transmission.^53^ Even in studies that involve endogenous peptide release, caged neuropeptides are valuable because they can be used to distinguish between changes in postsynaptic signaling (*i.e.*, in the receptor-expressing neuron) and the presynaptic, peptide-releasing cell. Moving *in vivo*, caged neuropeptides offer the ability to isolate the effects of time-resolved peptide signaling on neuronal activity and behavior.^54^ The ability to rapidly generate useful photopharmacology tools for neuropeptidergic systems will accelerate our understanding of neuropeptide signaling in the nervous system, which is currently an area of intense focus in neuroscience research.^55^

## Supporting Information Available

Materials and methods, detailed synthetic procedures, chemical analysis and spectra, and additional data and figures as noted in the text (PDF)

## Abbreviations

C-TEx: C-terminal extension
NPP: nitrophenyl peptide
DMNBA: dimethoxynitrobenzyl beta-alanine
GRP: gastrin-releasing peptide
GRPR: gastrin-releasing peptide receptor
OT: oxytocin
OTR: oxytocin receptor
SP: substance P
NK1R: neurokinin 1 receptor/tachykinin 1 receptor
CCK(8S): cholecystokinin octapeptide, sufated
CCK1R: cholecystokinin receptor 1
CCK2R: cholecystokinin receptor 2
VIP: vasoactive intestinal peptide
PV: parvalbumin
CIN: cholinergic interneuron
TLC: thin-layer chromatography
UV: ultraviolet
