# Supplementary material for: A Biomimetic C-Terminal Extension Strategy for Photocaging Amidated Neuropeptides
Source: J Am Chem Soc. 2023 Aug 31;145(36):19611–21. doi: 10.1021/jacs.3c03913 (PMC10510324; doi:10.1021/jacs.3c03913)
Supplement: Supplementary file 1 — ja3c03913_si_001.pdf [file ja3c03913_si_001.pdf]

Supporting Information

A biomimetic C-terminal extension strategy for photocaging amidated neuropeptides

Aryanna E. Layden, Xiang Ma, Caroline A. Johnson, Xinyi J. He, Stanley A. Buczynski, & Matthew R. Banghart

Table of Contents

Supporting figures 1-7……………………………………………………………………..………**S2-8**

Key resources table ……………………………………………………………………...…...**S9-11**

Chemical synthesis …………………………………………………………………….**S12-13**

NMR spectroscopy ……………………………………………………………………...**S14-17**

***In vitro* uncaging and dark stability**……...……………………………………………………**S18**

***In vitro* GPCR activation assays** ………………………………………………………**S18**

Brain slice preparation ………………………………………………………………………..**S19**

Electrophysiology ……………………………………………………………………………..**S19**

UV photolysis ……………………………………………………………………………………..**S19**

Data analysis ……………………………………………………………………………………...**S20**

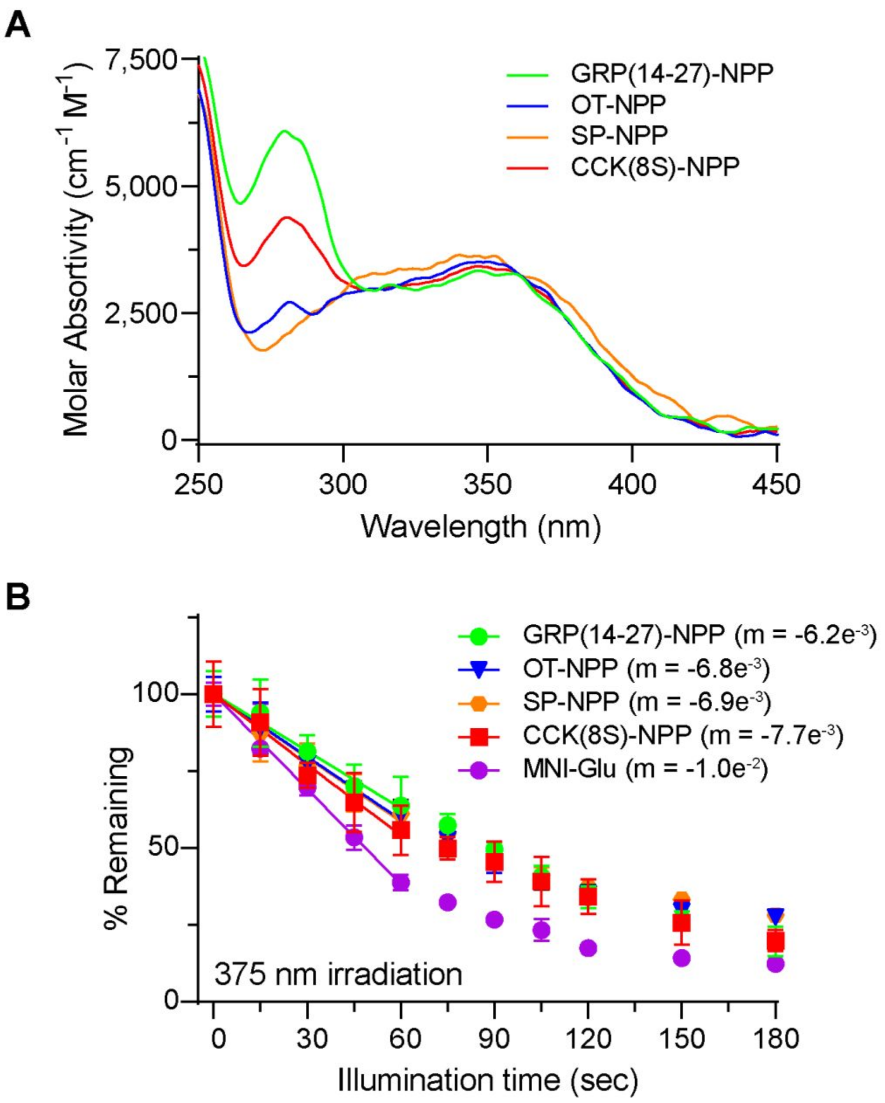

Supporting Figure 1. Photochemical characterization of NPP-caged neuropeptides. (**A**) UV/Vis absorbance spectra of GRP(14-27)-NPP, OT-NPP, SP-NPP, and CCK(8S)-NPP in phosphate buffered saline. (**B**) Plot of caged compound depletion over time in response to illumination with a 375 nm laser. Samples were optical density-matched at 375 nm in phosphate buffered saline. The percent of starting material remaining was determined by HPLC. Linear regression of data acquired during the first minute of illumination afforded a slope (% depletion/s) that reports the relative rates of photodegradation. MNI-glutamate was used as a reference compound.

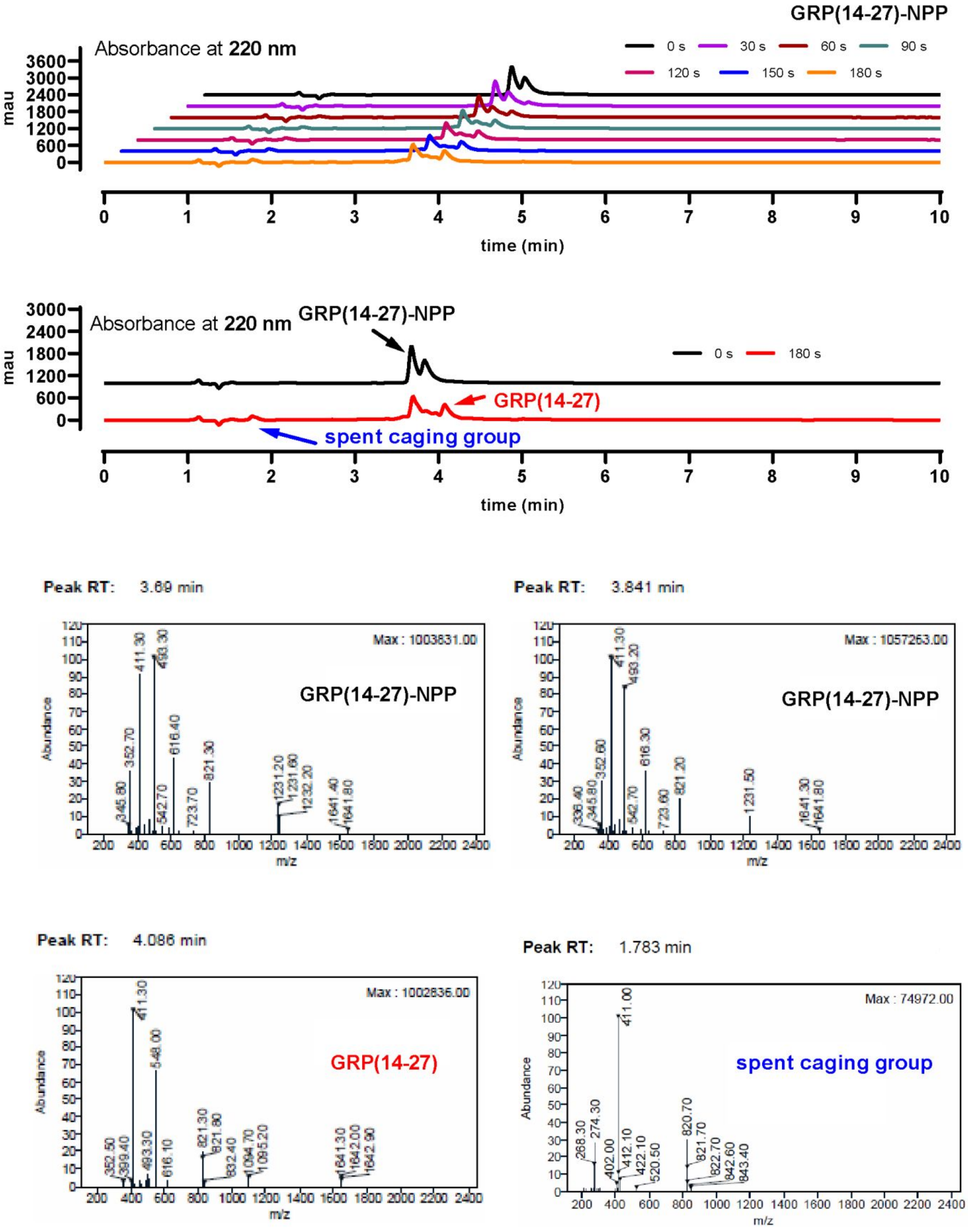

Supporting Figure 2. LC-MS analysis of GRP(14-27)-NPP photouncaging. (**A**) Waterfall plot of HPLC chromatograms monitoring the photoreaction over time. (**B**) Vertically offset chromatograms of the initial (0 sec) and final (180 sec) samples. (**C**) Mass spectrograms corresponding to the indicated peaks.

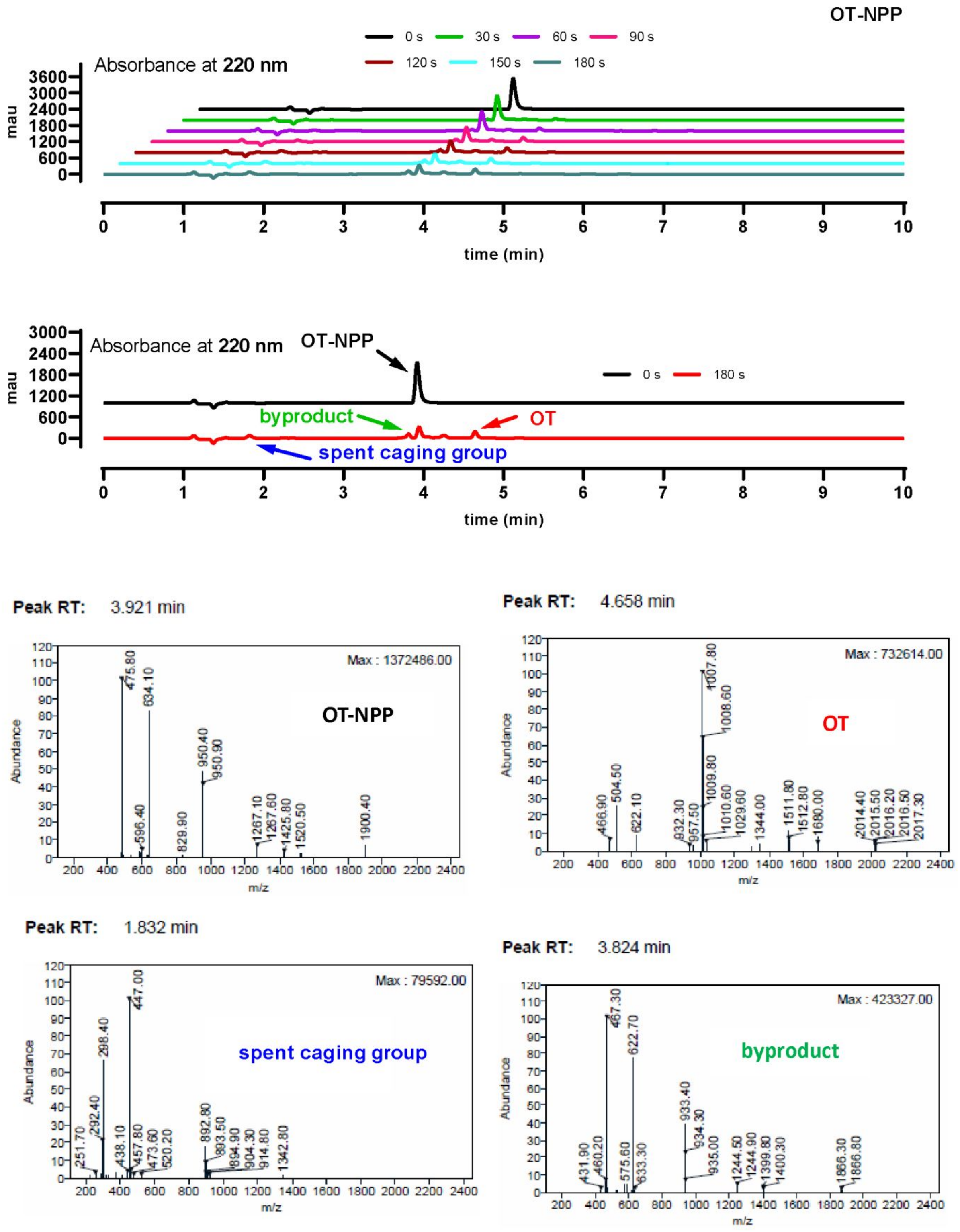

Supporting Figure 3. LC-MS analysis of OT-NPP photouncaging. (**A**) Waterfall plot of HPLC chromatograms monitoring the photoreaction over time. (**B**) Vertically offset chromatograms of the initial (0 sec) and final (180 sec) samples. (**C**) Mass spectrograms corresponding to the indicated peaks.

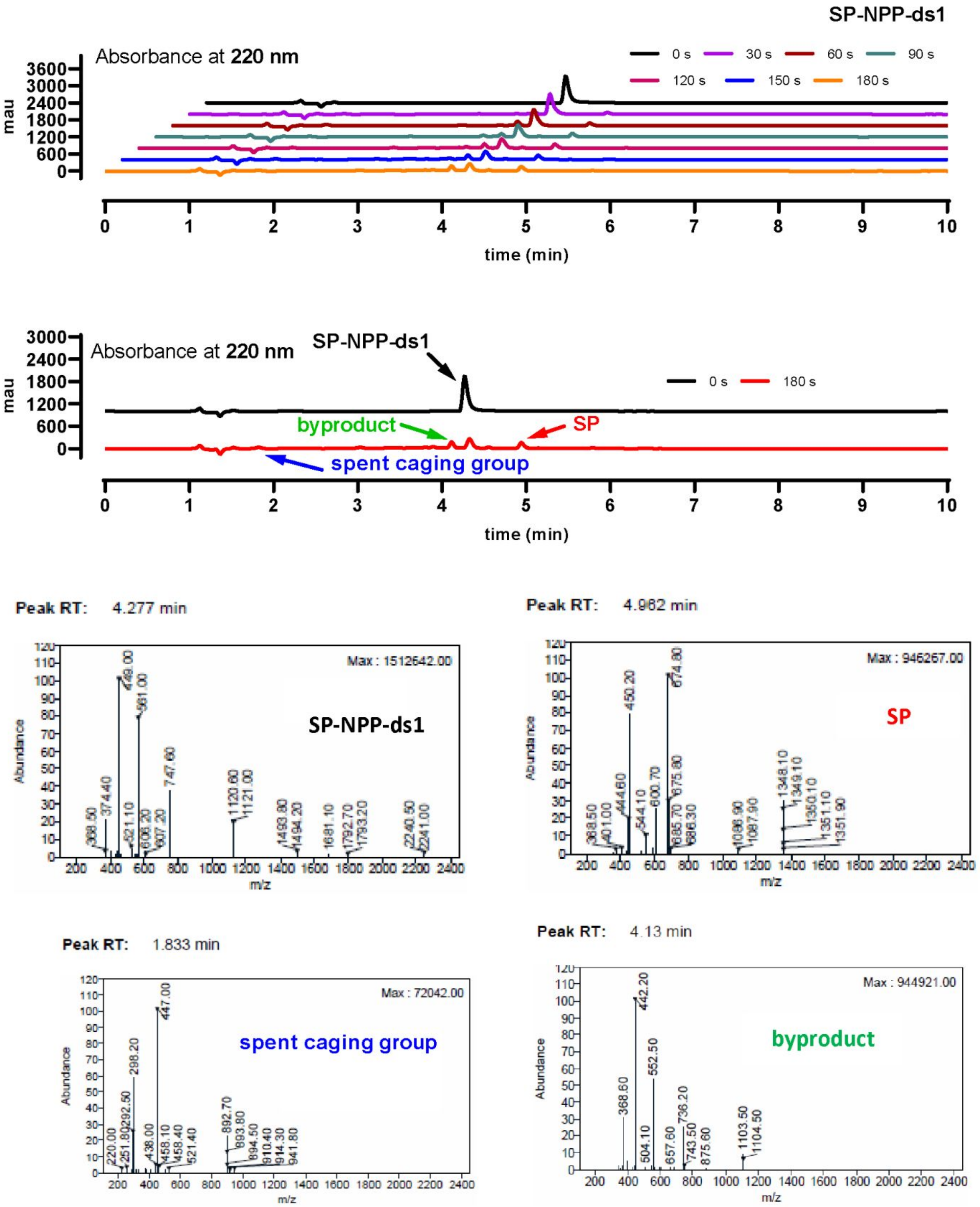

Supporting Figure 4. LC-MS analysis of SP-NPP-ds1 photouncaging. (**A**) Waterfall plot of HPLC chromatograms monitoring the photoreaction over time. (**B**) Vertically offset chromatograms of the initial (0 sec) and final (180 sec) samples. (**C**) Mass spectrograms corresponding to the indicated peaks.

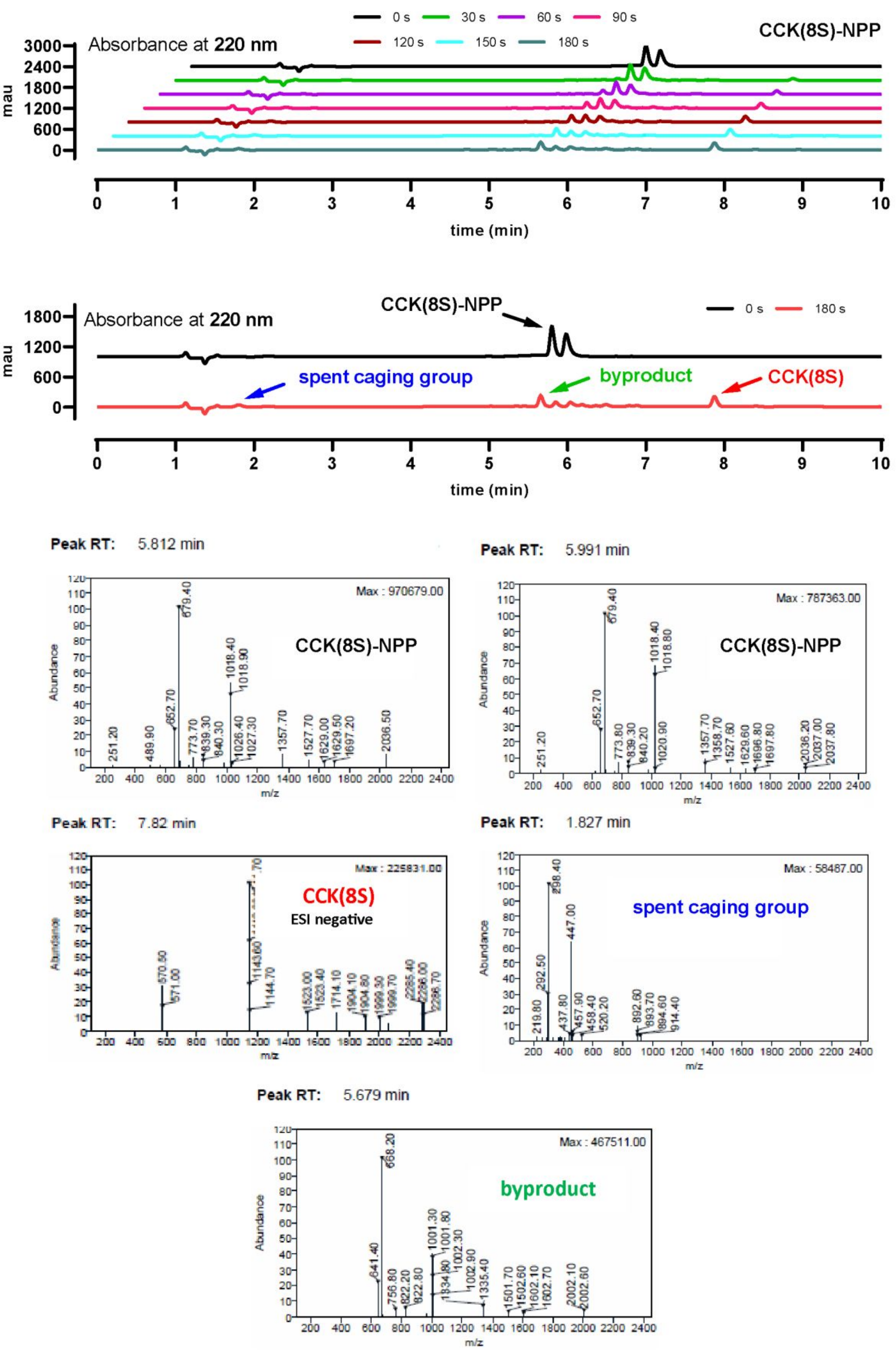

Supporting Figure 5. LC-MS analysis of CCK(8S)-NPP photouncaging. (**A**) Waterfall plot of HPLC chromatograms monitoring the photoreaction over time. (**B**) Vertically offset chromatograms of the initial (0 sec) and final (180 sec) samples. (**C**) Mass spectrograms corresponding to the indicated peaks.

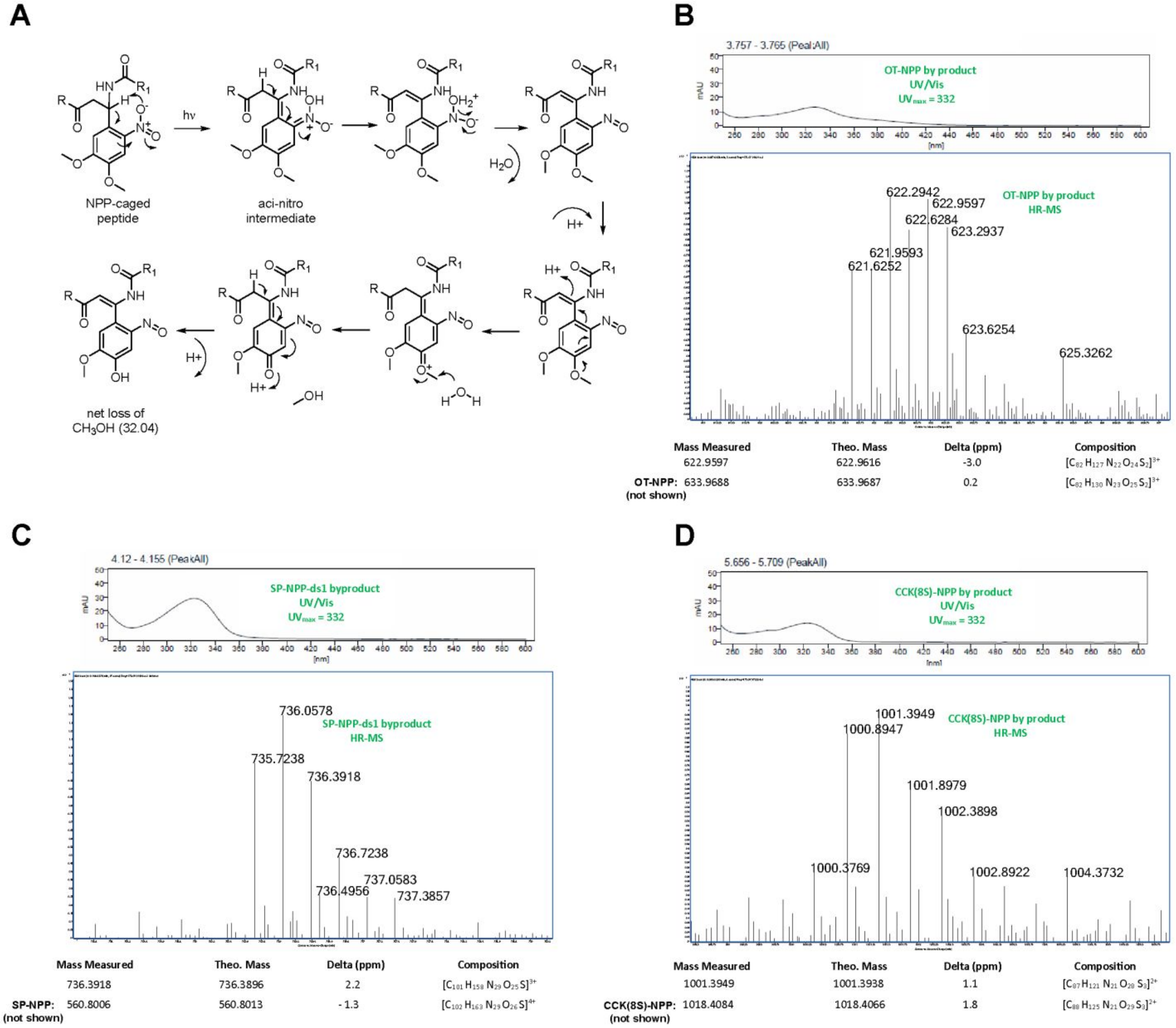

Supporting Figure 6. Characterization of the photoreaction byproduct. (**A**) Proposed mechanism and structure of the byproduct. (**B**) (Top) UV/Vis spectrum extracted from HPLC analysis of the photoreaction and (bottom) HR-MS spectrogram of the byproduct of OT-NPP photolysis. (**C**) Same as B but for SP-NPP-ds1. (**D**) Same as B but for CCK(8S)-NPP.

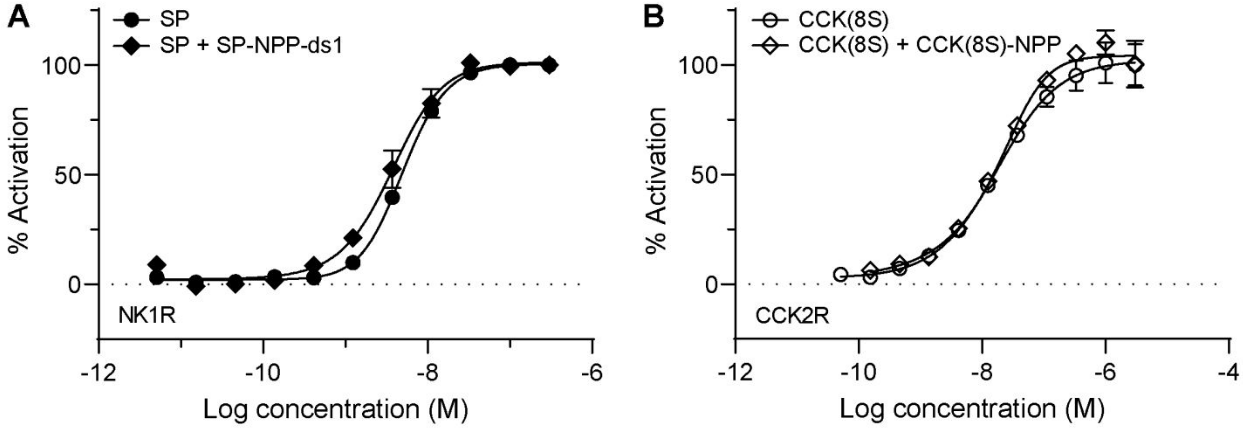

Supporting Figure 7. *In vitro* assessment of antagonism using the GloSensor assay of cAMP signaling. (**A**) Dose-response curves for substance P (SP) at the neurokinin 1 receptor (NK1R) in the absence (EC50 = 5 nM) and presence (EC50 = 3.7 nM) of SP-NPP-ds1 (1 µM) (n=10 wells per data point). Data were normalized to the maximal response to SP (0.3 µM) and are expressed as the mean ± SEM. (**B**) Same as A, but for CCK(8S) at the cholecystokinin 2 receptor (CCK2R) in the absence (EC50 = 17.6 nM) and presence (EC50 = 17.3 nM) of CCK(8S)-NPP (1 µM). Data were normalized to the maximal response to CCK(8S) (3 µM) and are expressed as the mean ± SEM.

Key resources table

Reagent type (species) or resource	Designation	Source or reference	Identifiers
GRPR plasmid	mGRPR	Genscript	Clone ID OMu14220D
OTR plasmid	hOXR	cdna.org	Cat # OXTR000000
CCK1R plasmid	hCCK1R	cdna.org	Cat # CCKAR00000
CCK2R plasmid	hCCK2R	cdna.org	Cat # CCKBR00000
NK1R plasmid	hNK1R	cdna.org	Cat # TACR100000
HEK293T cells		ATCC.org	Cat # CRL-3216
GloSensor plasmid	pGloSensor - 22F	Promega	Cat # E2301
GloSensor reagent	GloSensor cAMP Reagent (luciferin)	Promega	Product # E1291
VIP-IRES-CRE, C57Bl/6J background (*M. musculus*, male and female)	*Vip*^{Cre}	The Jackson Laboratory	Cat # 010908
Ai14, C57Bl/6J background (*M. musculus*, male and female)	*Rosa26-lsl-tdTomato* (Ai14)	The Jackson Laboratory	Cat # 007914
ChAT-IRES-CRE, C57Bl/6J background (*M. musculus*, male and female)	*Chat*^{Cre}	The Jackson Laboratory	Cat # 006410
Pvalb-IRES-Cre, C57Bl/6J background (*M. musculus*, male and female)	*Pvalb*^{Cre}	The Jackson Laboratory	Cat # 017320
Gastrin-releasing peptide (14-27)	GRP(14-27)	BaChem	Product Number: 4011693<br><br>CAS Number: 93755-85-2

Cholecystokinin Octapeptide (sulfated)	CCK(8S)	BaChem	Product Number: 4033010<br><br>CAS Number: 25126-32-3
Oxytocin	OT	BaChem	Product Number: 4016373<br><br>CAS Number: 50-56-6
Substance P	SP	BaChem	Product Number: 4010600<br><br>CAS Number: 33507-63-0
2,3-Dioxo-6-nitro-1,2,3,4-tetrahydrobenzo[*f*]quinoxaline-7-sulfonamide disodium salt	NBQX	HelloBio	Cat # HB0443
3-((*R*)-2-Carboxypiperazin-4-yl)-propyl-1-phosphonic acid	(R)-CPP	HelloBio	Cat # HB0021
Picrotoxin	Picrotoxin	Sigma	Cat # P1675
3-Phenylpropanoyl-His-Trp-Ala-Val-D-Ala-His-D-Pro-CH2NH-Phe-NH2	BW2258U89	Phoenix Pharmaceuticals, Inc.	Product # 027-22
d$(CH_2)_5^1$,Tyr(Me)^2,Thr^4,Orn^8,des-Gly-NH_2^9)-Vasotocin Trifluoroacetate	Oxytocin antagonist (OTA)	BaChem	Product # 4031339
(*R*)-*N*-[2,3-Dihydro-1-[2-(2-methylphenyl)-2-oxoethyl]-2-oxo-5-phenyl-1*H*-1,4-benzodiazepin-3-yl]-*N'*-(3-methylphenyl)-urea	YM 022	Tocris	Cat # 1408
(3a*R*,7a*R*)-Octahydro-2-[1-imino-2-(2-methoxyphenyl)ethyl]-7,7-diphenyl-4*H*-isoindol	RP67580	Tocris	Cat # 1635
Phosphoramidon disodium salt	Phosphoramidon	Tocris	Cat # 6333
Bestatin	Bestatin	Tocris	Cat # 1956

Butabindide oxalate	Butabindide	Santa Cruz Bio	Cat # sc361130
[Leu]^5-enkephalin	Enk	Tocris	Cat # 1889
4-Methoxy-7-nitroindolinyl-caged-L-glutamate	MNI-glutamate	Tocris	Cat # 1490
Software	MatLab 2009B	Mathworks Inc	RRID:SCR_001622
Software	ScanImage	Pologruto, T. A.; Sabatini, B. L.; Svoboda, K. *Biomed. Eng. Online* **2003**, *2*.	RRID:SCR_014307
Software	Igor Pro 6.02A	WaveMetrics	RRID:SCR_000325
Software	GraphPad Prism 9.5.1	GraphPad Inc	RRID:SCR_002798
Software	Excel (2016)	Microsoft	RRID:SCR_016137
Software	ChemDraw Professional 20.1.1.125	PerkinElmer	
Software	Adobe Illustrator 27.4	Adobe Systems Inc	RRID:SCR_010279

Chemical Synthesis

Large-scale synthesis of racemic Fmoc-DMNBA (**4**) and the synthesis of all four NPP-caged peptides was conducted by BaChem. NPP-caged peptides were purified by high-pressure liquid chromatography and their identities confirmed by high resolution mass spectrometry (HRMS). Diastereomers were observed in ~equal proportion and were readily resolved for SP-NPP but not for GRP(14-27)-NPP, OT-NPP, and CCK(8S)-NPP.

Commercial reagents were used as received. All solvents were purchased as septum-sealed bottles stored under an inert atmosphere. All reactions were sealed with septa through which a nitrogen atmosphere was introduced unless otherwise noted. Reactions were conducted in round-bottomed flasks or septum-capped amber screw-cap vials containing Teflon-coated magnetic stir bars. Reactions were monitored by liquid chromatography-mass spectrometry (Agilent 1260 Infinity II) using C-18 column (4.6 × 50 mm, 1.8 μm, Agilent) with a linear gradient (water/MeCN 5%/95% → MeCN 100%, 0-8 min with 0.1% formic acid, 1 ml/min flow, electrospray ionization, positive ion mode, UV detection at 220 nm, 280 nm, and 350 nm). High-resolution mass spectrometry data were obtained at the UCSD Chemistry and Biochemistry Mass Spectrometry Facility on an Agilent 6230 time-of flight mass spectrometer (TOFMS). Proton (^{1}H) and carbon (^{13}C) NMR spectra were recorded at room temperature in DMSO, MeCN, D2O or base-filtered CDCl3 on a Bruker AVA-400, Varian VX-500, or Jeol ECA-500 spectrometer operating at 400 MHz for proton and 100 or 101 MHz for carbon nuclei. For ^{1}H NMR spectra, signals arising from the residual protioforms of the solvent were used as the internal standards. ^{1}H NMR data are reported as follows: chemical shift (*δ*) [multiplicity, coupling constant(s) *J* (Hz), relative integral] where multiplicity is defined as: s = singlet; d = doublet; t = triplet; q = quartet; m = multiplet or combinations of the above. All NMR spectra were processed using MestReNova 14.2.1. UV-visible spectra were recorded on a NanoDrop 2000 UV-VIS spectrophotometer (Thermo-Fisher). Room lights were covered with Roscolux Canary Yellow #312 film (Rosco Laboratories, Stamford, CT) to filter out wavelengths of light that could lead to unintentional photolysis during purification and handling.

3-amino-3-(4,5-dimethoxy-2-nitrophenyl)propanoic acid (1). 4,5-dimethoxy-2-nitrobenzaldehyde (8.55 g, 51.4 mmol, 1 eq) was dissolved in EtOH (100 mL) followed by the addition of NH_4OAc (11.90 g, 154.4 mmol, 3 eq), and malonic acid (10.71 g, 60.18 mmol, 2 eq). The reaction mixture was refluxed overnight (18 hours) to find a milky thick white solution. This solution was then filtered and washed with EtOH until the filtrate was colorless. Solvent removal afforded 3-amino-3-(4,5-dimethoxy-2-nitrophenyl)propanoic acid as a white solid. Yield: 92%. 1H NMR (400 MHz, D2O_K2CO3) δ 7.11 – 6.93 (m, 3H), 4.26 (t, J = 7.3 Hz, 1H), 3.88 (d, J = 1.5 Hz, 3H), 3.85 (dd, J = 5.7, 1.4 Hz, 3H), 3.11 (d, J = 1.4 Hz, 2H), 2.67 – 2.50 (m, 2H). 13C NMR (101 MHz, D2O_NaOH) δ 180.19, 147.94, 147.01, 137.86, 118.95, 111.79, 110.06, 55.69, 55.61, 52.68, 46.98. ESI-HRMS (+): m/z calcd: 225.1001; found: 226.1077 [M+H].

methyl 3-(3,4-dimethoxyphenyl)-3-(2,2,2-trifluoroacetamido)propanoate (2). 3-amino-3-(4,5-dimethoxy-2-nitrophenyl)propanoic acid (1.13 g, 5.02 mmol, 1 eq) was placed in a round bottom flask, sealed and purged with nitrogen 3 times before dissolving in trifluoroacetic anhydride (21.0 mL, 150.5 mmol, 30 eq) and mixed vigorously (1 hour) or sonicated until the reaction mixture becomes fully homogenous. This mixture was then cooled down to 0°C

followed by the addition of acetyl chloride (3.6 ml, 50.17 mmol, 10 eq) and slow dropwise addition of anhydrous methanol (6.09 ml, 150.5 mmol, 30 eq). The reaction mixture was allowed to stir for 1 hour before quenching with water (500 mL) followed by 3 washes with CH_2Cl_2. The organic layers were combined, concentrated in vacuo, and purified by silica gel chromatography (0-30% EtOAc in hexane for 10 column volumes) to give methyl 3-(3,4-dimethoxyphenyl)-3-(2,2,2-trifluoroacetamido)propanoate as a white oil. Yield: 45%. 1H NMR (400 MHz, CHLOROFORM-D) δ 7.66 (d, J = 8.3 Hz, 1H), 6.84 – 6.79 (m, 2H), 6.78 (d, J = 1.6 Hz, 1H), 5.35 (dt, J = 8.4, 5.6 Hz, 1H), 3.86 (s, 3H), 3.85 (s, 3H), 3.65 (s, 3H), 2.93 (qd, J = 16.1, 5.6 Hz, 2H). 13C NMR (101 MHz, CHLOROFORM-D) δ 171.66, 149.33, 149.06, 131.10, 118.23, 111.36, 109.68, 56.00, 52.29, 49.93, 38.94. ESI-HRMS (+): m/z calcd: 335.0981; found: 336.1058 [M+H].

methyl 3-(4,5-dimethoxy-2-nitrophenyl)-3-(2,2,2-trifluoroacetamido)propanoate (3). Methyl 3-(3,4-dimethoxyphenyl)-3-(2,2,2-trifluoroacetamido)propanoate (232 mg, 692 µmol, 1 eq) was placed in a round bottom flask, sealed and purged with nitrogen 3 times before cooling down the flask to 0°C. Nitric acid (6.0 ml, 134 mmol, 194 eq) was slowly added to the reaction mixture which changed to an orange color. The reaction mixture was covered in tin foil and kept away from light for 2 hours while stirring. Afterward water (100 ml) was added the reaction mixture and the reaction left overnight at 6ºC. In the morning the reaction was filtered with copious amounts of water until the filtrate was clear, followed by drying to give methyl 3-(4,5-dimethoxy-2-nitrophenyl)-3-(2,2,2-trifluoroacetamido)propanoate as a white solid. Yield: 80.9%. 1H NMR (400 MHz, DMSO-D6) δ 10.10 (d, J = 7.9 Hz, 1H), 7.53 (s, 1H), 7.23 (s, 1H), 5.83 (ddd, J = 10.2, 7.8, 4.1 Hz, 1H), 3.83 (s, 3H), 3.81 (s, 3H), 3.61 (s, 3H), 3.06 – 2.84 (m, 2H). 13C NMR (126 MHz, DMSO-D6) δ 170.01, 153.25, 147.88, 140.16, 129.87, 115.99, 113.69, 109.97, 107.65, 56.30, 56.14, 51.80, 46.07, 38.87. ESI-HRMS (+): m/z calcd: 380.0831; found: 381.0904 [M+H].

3-((((9H-fluoren-9-yl)methoxy)carbonyl)amino)-3-(4,5-dimethoxy-2-nitrophenyl)propanoic acid (4). Methyl 3-(4,5-dimethoxy-2-nitrophenyl)-3-(2,2,2-trifluoroacetamido)propanoate (23.2 mg, 61.0 µmol, 1 eq) was mixed with of 2M NaOH (0.5 mL)and stirred for 15 minutes. Deprotected product was verified on TLC and reaction mixture neutralized before removing excess water in vacuo. DIPEA (31.9 µl, 183 µmol, 3 eq), (9H-fluoren-9-yl)methyl (2,5-dioxopyrrolidin-1-yl) carbonate (41.2 mg, 122 µmol, 2 eq), and 1 ml DMF were added to the reaction mixture and this stirred for 5 hours or until all starting material was consumed. The reaction mixture was then mixed with water and washed with EtOAc. The organic layer was then concentrated in vacuo and purified by silica gel chromatography (0-5% methanol in CH_2Cl_2) to give 3-((((9H-fluoren-9-yl)methoxy)carbonyl)amino)-3-(4,5-dimethoxy-2-nitrophenyl)propanoic acid as an off-white solid. Yield 78%. 1H NMR (400 MHz, MeCN-D3) δ 7.83 – 7.76 (m, 2H), 7.58 (t, J = 6.6 Hz, 2H), 7.49 (s, 1H), 7.37 (s, 3H), 7.26 (q, J = 7.0 Hz, 2H), 7.03 (s, 1H), 6.53 (d, J = 8.1 Hz, 1H), 5.60 (td, J = 9.2, 4.1 Hz, 1H), 4.38 – 4.09 (m, 3H), 3.87 (s, 3H), 3.84 (s, 3H), 2.88 – 2.73 (m, 2H). 13C NMR (100 MHz, DMSO-D6) δ 171.97, 155.86, 153.74, 147.91, 145.75, 144.48, 144.07, 141.27, 141.23, 140.43, 128.15, 127.68, 127.53, 127.45, 127.31, 125.77, 125.61, 120.64, 120.39, 107.81, 65.97, 64.32, 56.76, 56.55, 48.13, 47.15.ESI-HRMS (+): [M+H] 493.48 [M+23] 515.48. ESI-HRMS (+): m/z calcd: 492.15327; found: 493.1605 [M+H].

NMR spectroscopy

3-amino-3-(4,5-dimethoxy-2-nitrophenyl)propanoic acid (1)

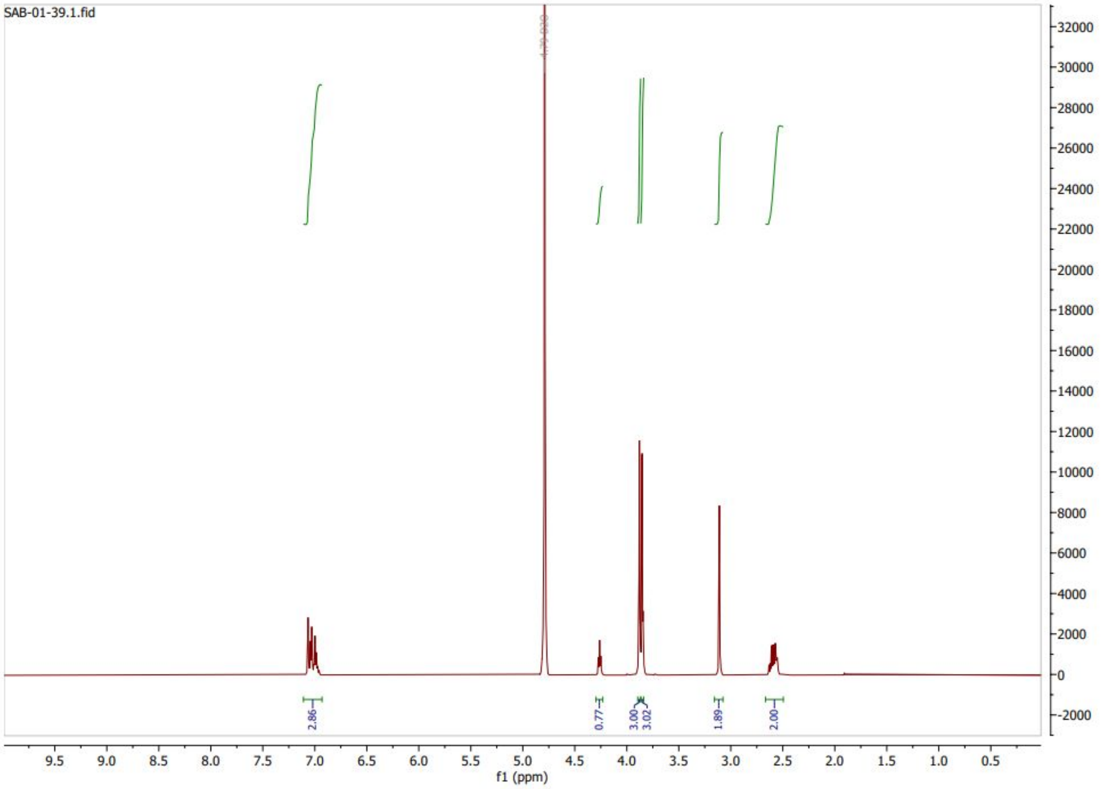

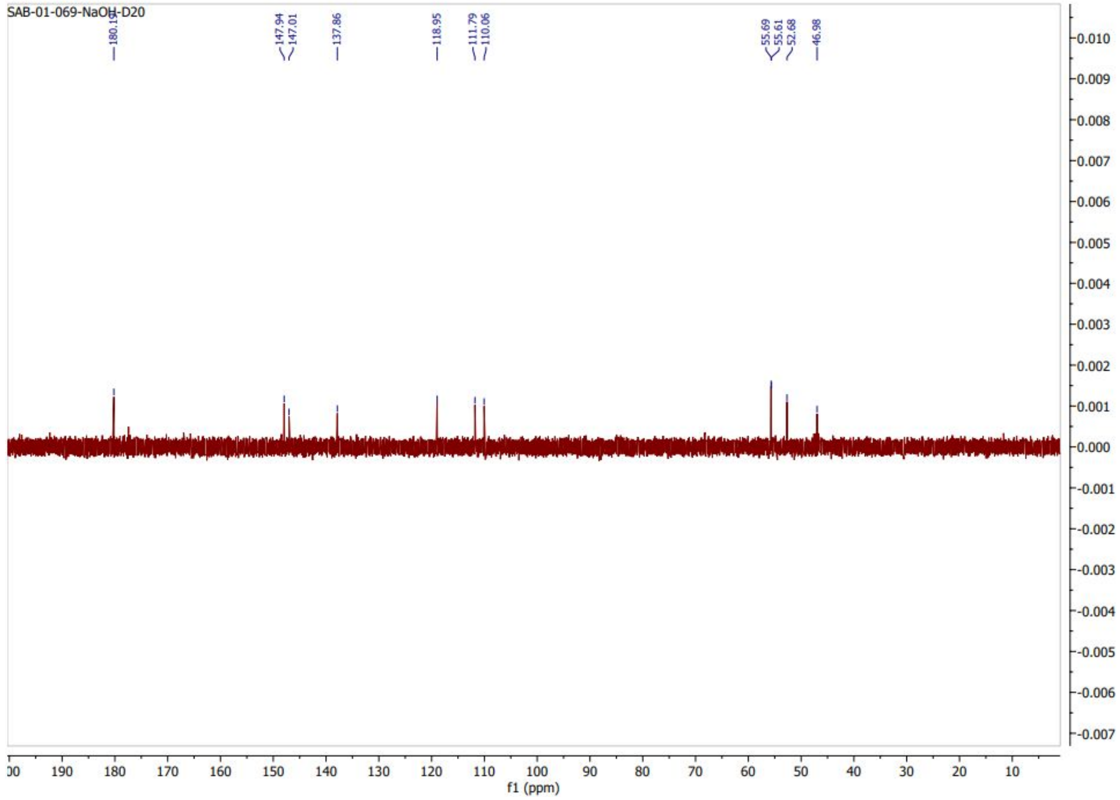

Methyl 3-(3,4-dimethoxyphenyl)-3-(2,2,2-trifluoroacetamido)propanoate (2)

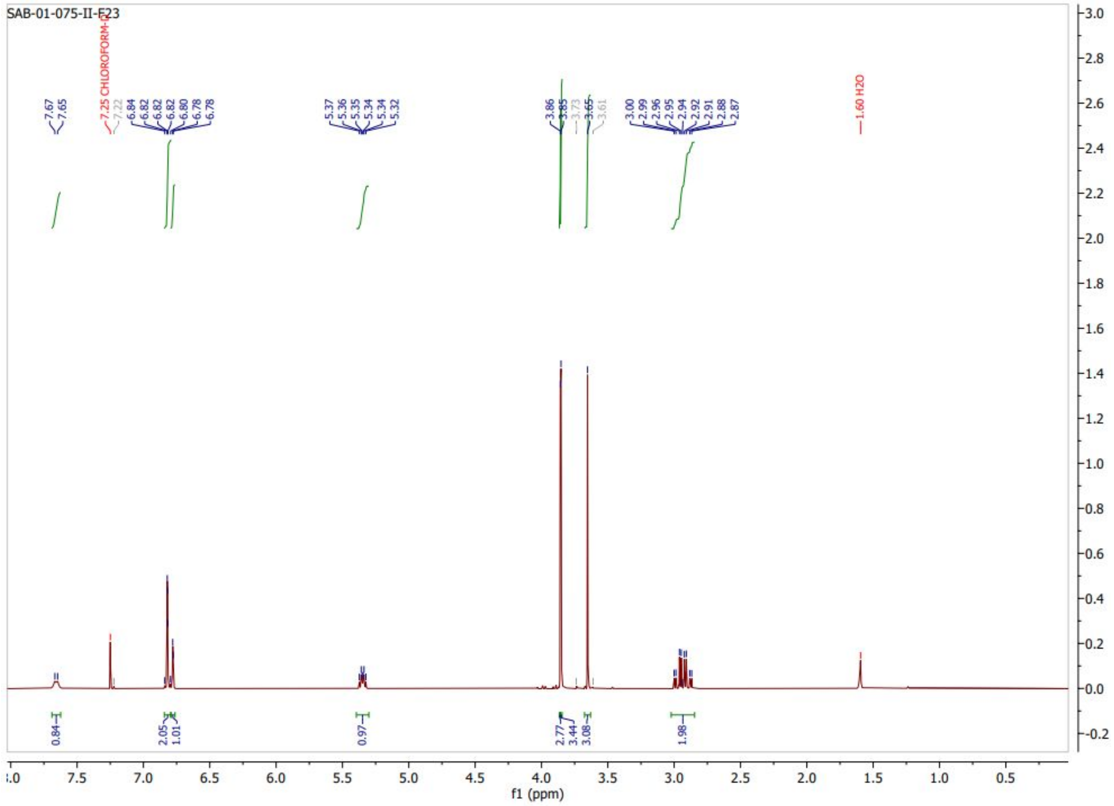

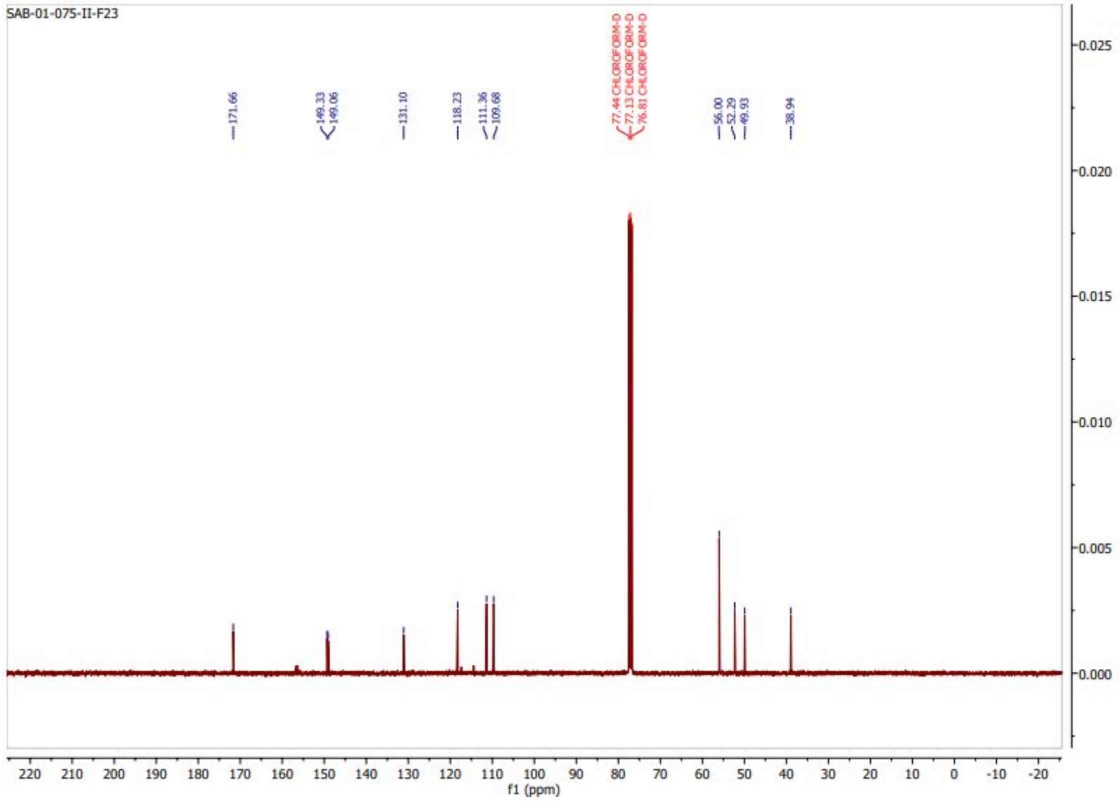

Methyl 3-(4,5-dimethoxy-2-nitrophenyl)-3-(2,2,2-trifluoroacetamido)propanoate (3)

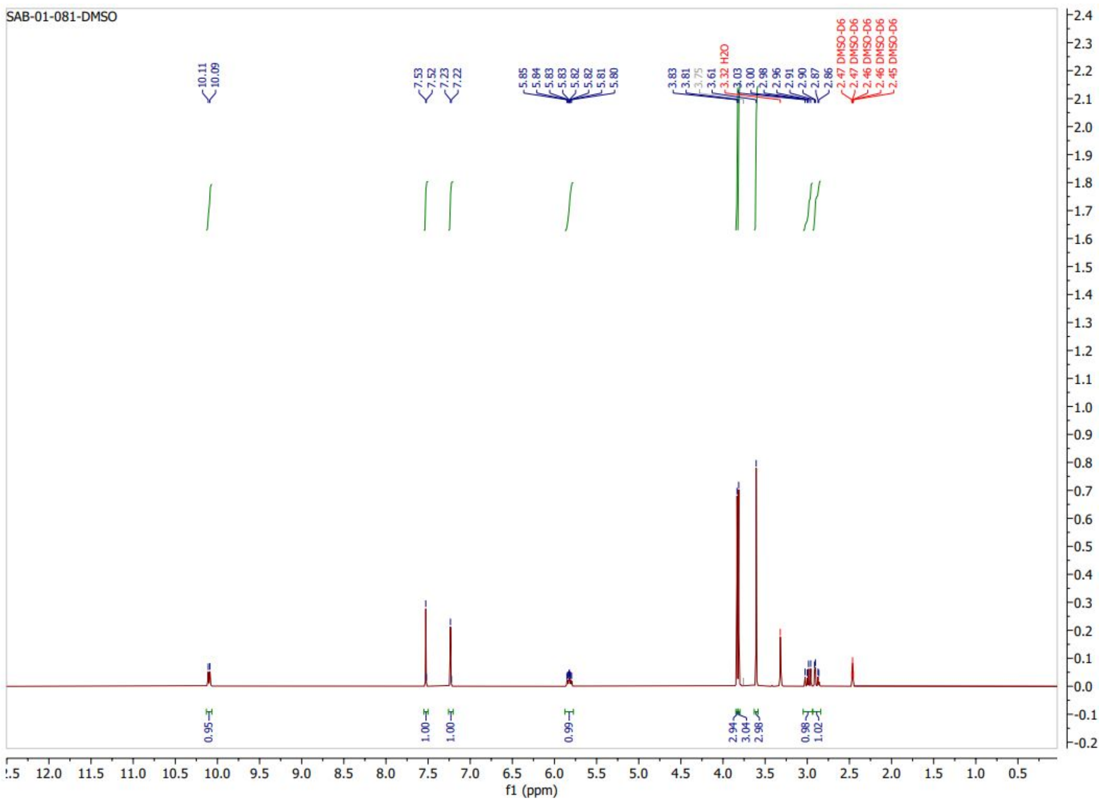
SAB-01-081-DMSO
f1 (ppm)

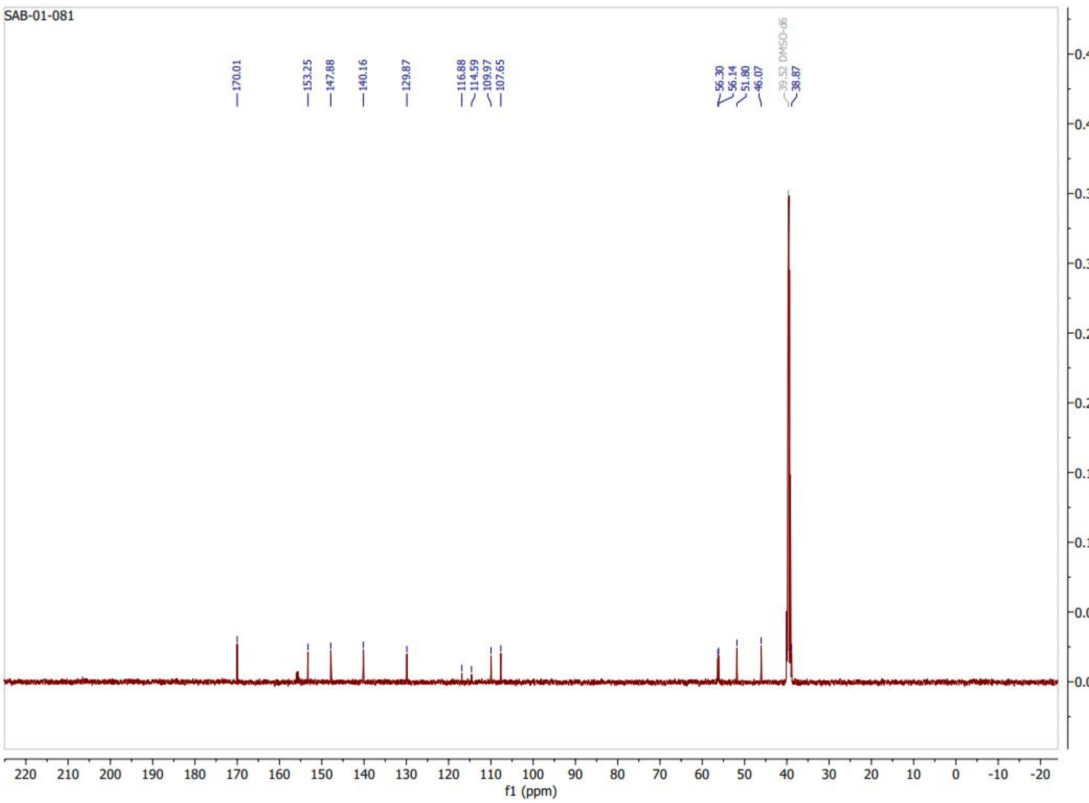
SAB-01-081
f1 (ppm)

3-((((9H-fluoren-9-yl)methoxy)carbonyl)amino)-3-(4,5-dimethoxy-2-nitrophenyl)propanoic acid (4)

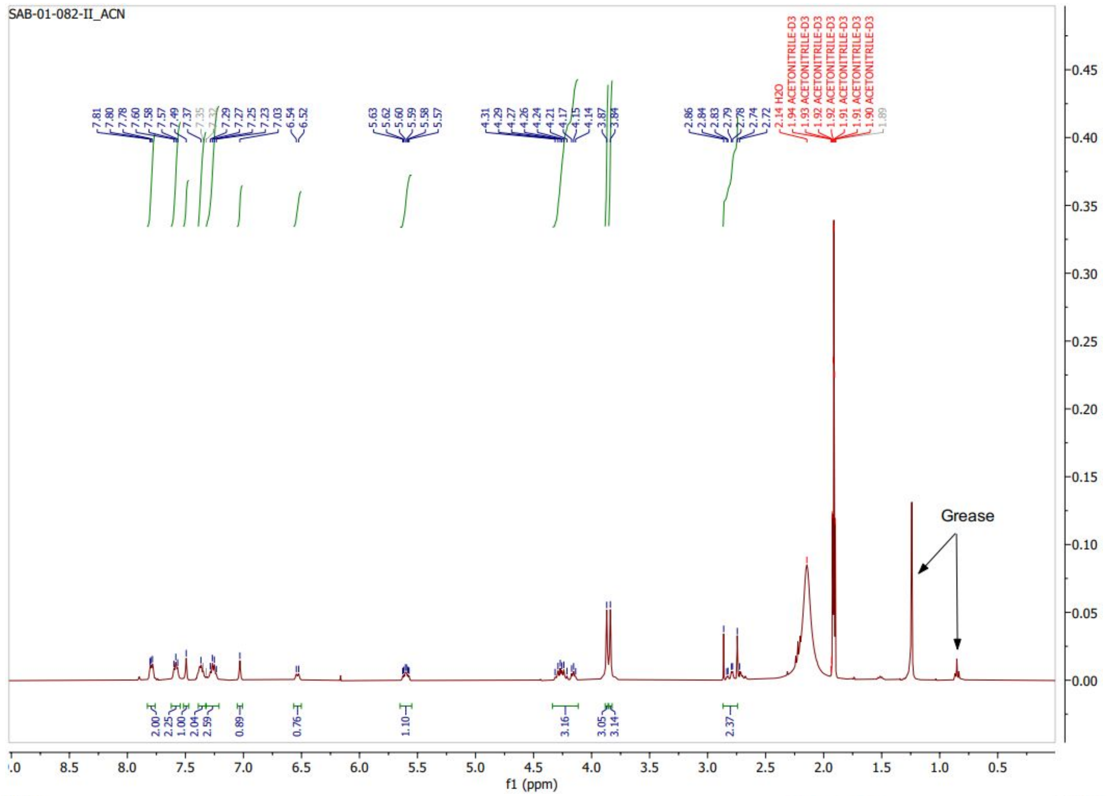

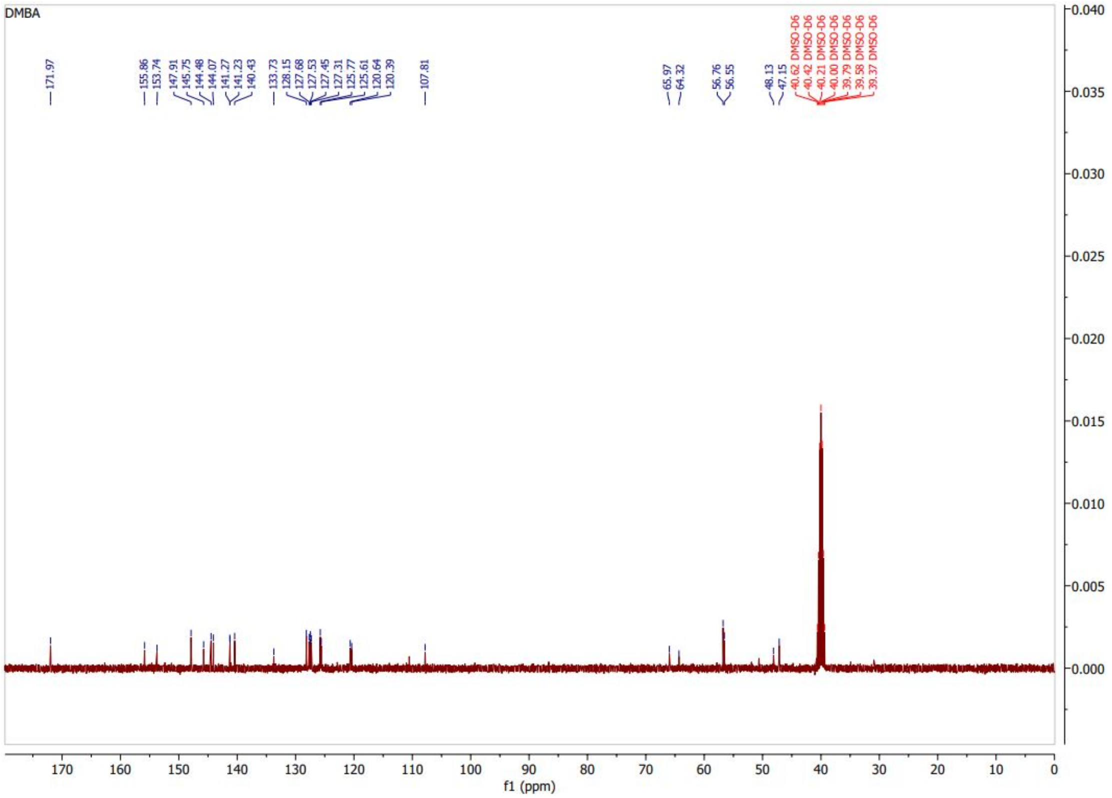

***In vitro* uncaging and dark stability.** To determine dark stability the NPP-caged peptides were dissolved in phosphate-buffered saline (PBS, pH 7.2) at a concentration of 1 mM and left in the dark for 24 h. Comparison of samples taken at 0 and 24 h by HPLC-MS (1260 Affinity II, Agilent Technologies, Santa Clara, CA, USA) revealed no obvious decomposition or conversion to the parent peptide. In addition to determining the chemical composition of the uncaging products by LC-MS, the initial photolysis rate for each NPP-caged peptide in response to illumination with a 375 nm laser was compared to MNI-Glutamate using HPLC. The concentrations of the NPP-caged peptides and MNI-glutamate were adjusted to match their optical densities at the photolysis wavelength of 375 nm. Solutions of NPP-caged peptides (0.4 mM) and MNI-glutamate (0.5 mM) dissolved in PBS buffer (pH 7.2) were placed in 1 mL glass vials with stir bars and illuminated at a light intensity of 10 mW from a 375 nm laser (LBX-375-400-HPE-PPA, Oxxius, France) via an optical fiber (FT200UMT, 200 µm, 0.39 NA). The solutions were illuminated in 15 sec periods, after which samples were removed and analyzed by LC-MS using a linear gradient (water/MeCN 5%/95% → MeCN 100%, 0-8 min with 0.1% formic acid) and a C-18 column (4.6 × 50 mm, 1.8 µm) (Agilent). Each compound was assessed in triplicate. The integrals of the remaining caged molecule from each sample were normalized to the integral of the un-illuminated sample, averaged and plotted against time for the first two minutes of illumination. Linear regression provided a measurement of slope, and the ratio of the two slopes relates to the relative photolysis efficiency of the NPP-caged peptides in comparison to MNI-glutamate.

***In vitro* GPCR activation assays.** GloSensor assay of G-protein signaling. Human embryonic kidney 293T cells were grown in Complete DMEM (Dulbecco's modified Eagle's medium (Invitrogen, Carlsbad, CA) containing 5% fetal bovine serum (Corning), 50 U/mL Penicillin-Streptomycin (Invitrogen), and 1 mM sodium pyruvate (Corning)) and maintained at 37 °C in an atmosphere of 5% CO_2 in 10 cm TC dishes. Media in 10 cm TC dishes with HEK 293T cells (at around 70% confluence) were replaced with Opti-MEM (Invitrogen). Then the GPCR-plasmid, cAMP dependent reporter plasmid (pGloSensor -22F), and Lipofectamine 2000 (Invitrogen) in Opti-MEM were added. The dishes with transfection media were incubated at 37°C in an atmosphere of 5% CO_2 for 6 h before replacing the media with complete DMEM. After incubating at 37°C in an atmosphere of 5% CO_2 for 16 h, transfected cells were plated in poly-*D*-lysine coated 96-well plates at ~40,000 cells/well and incubated at 37 °C in an atmosphere of 5% CO_2 for 16 h. On the day of assay, media in each well were replaced with 50 µL of assay buffer (20 mM HEPES, 1x HBSS, pH 7.2, 2 g/L d-glucose), followed by addition of 25 µL of 4x drug solutions for 15 min at room temperature. Subsequently, 25 µL of 4 mM GloSensor cAMP Reagent (luciferin) was added, and, following gentle mixing, luminescence counting was performed using a plate reader (iD5, Molecular Devices) after 25 min. For antagonism experiments, the candidate antagonist was added to the assay buffer (50 uL/well) at 2x the final concentration and allowed to incubate for 5 minutes prior to the addition of agonist.

Brain slice preparation. All procedures were performed in accordance with protocols approved by the University of California San Diego Institutional Animal Care and Use Committee (IACUC) following guidelines described in the the US National Institutes of Health Guide for Care and Use of Laboratory Animals (UCSD IACUC protocol S16171). Experiments were conducted using mice of both sexes and the data were combined. From *Vip*^{Cre}; *Rosa26-lsl-tdTomato* (Ai14), mouse age ranged from P15-30. From *Pvalb*^{Cre}; *Rosa26-lsl-tdTomato* (Ai14), mouse age ranged from P20-35. From *Chat*^{Cre}; *Rosa26-lsl-tdTomato* (Ai14), mouse age ranged from P40-100. Mice were anesthetized with isoflurane and decapitated, and the brain was removed, blocked, and mounted in a VT1000S vibratome (Leica Instruments). For recordings from cortex and striatum, coronal slices (300 µm) were prepared, and for hippocampus, horizontal slices were prepared in ice-cold choline-ACSF containing (in mM) 25 NaHCO3, 1.25 NaH2PO4, 2.5 KCl, 7 MgCl2, 25 glucose, 0.5 CaCl2, 110 choline chloride, 11.6 ascorbic acid, and 3.1 pyruvic acid, equilibrated with 95% O2/5% CO2. Slices were transferred to a holding chamber containing oxygenated artificial cerebrospinal fluid (ACSF) containing (in mM) 127 NaCl, 2.5 KCl, 25 NaHCO3, 1.25 NaH2PO4, 2 CaCl2, 1 MgCl2, and 10 glucose, osmolarity 290. Slices were incubated at 32 °C for 30 min and then left at room temperature until recordings were performed.

Electrophysiology. All recordings were performed within 5 h of slice cutting in a submerged slice chamber perfused with ACSF warmed to 32 °C and equilibrated with 95% O2/5% CO2. Whole-cell voltage clamp recordings were made with an Axopatch 700B amplifier (Axon Instruments). Data were filtered at 3 kHz, sampled at 10 kHz, and acquired using National Instruments acquisition boards and a custom version of ScanImage[46] written in MATLAB (Mathworks). Cells were rejected if holding currents exceeded −200 pA or if the series resistance (<25 MΩ) changed during the experiment by more than 20%. Patch pipets (open pipet resistance 2.0−3.0 MΩ) were filled with an internal solution containing (in mM) 135 KMeSO4, 5 KCl, 5 HEPES, 1.1 EGTA, 4 MgATP, 0.3 Na_2GTP, and 10 Na_2phosphocreatine (pH 7.25, 286 mOsm/kg). Synaptic transmission was blocked with the addition to the ACSF of 2,3-dihydroxy-6-nitro-7-sulfamoyl-benzo(f)quinoxaline (NBQX; 10 µM), R,S-3-(2-carboxypiperazin-4-yl)propyl-1-phosphonic acid (CPP; 10 µM), and picrotoxin (10 µM). Recordings were targeted to TdTomato-expressing neurons visualized through a Cy3 filter cube (Semrock Cy3-4040C). Motor cortex VIP-neurons were held at −55 mV, hippocampal PV-expressing neurons at -70 mV, and striatal cholinergic interneurons -60 mV.

UV photolysis. For experiments with GRP(14-27)-NPP, OT-NPP, and CCK(8S)-NPP, uncaging was carried out using flashes of collimated full-field illumination with a 355 nm laser (DPSS Lasers), delivered through a 60x LUMPLANFL 1.0 NA objective (Olympus) on SliceScope Pro 6000 microscope (Scientifica) using a custom uncaging path as previously described[3]. Light power ranged from 50-80 mW and corresponds to measurements of a 10 mm diameter collimated beam at the back aperture of the objective. Flash duration ranged from 20-200 ms, as reported in the text. Beam size exiting the objective onto the sample was 3,900 μm^2. For experiments with SP-NPP, uncaging was achieved using full-field illumination from the 365 nm-UV channel of a pE-300white LED (CoolLED) reflected off a 405 nm long-pass dichroic mirror (Di02-R405-25x36, Semrock) mounted in the fluorescence turret on the same microscope. Light

power was set to 5 mW in the sample plane (~80 mW of an ~20 mm diameter "beam" at the back aperture).

Data analysis. Electrophysiology data were analyzed in Igor Pro (Wavemetrics). Current response to bath application of peptide were calculated as the average current over a 20 sec window surrounding the peak current measured for each cell. For each parent peptide, peak responses were obtained at different time windows after peptide addition (GRP(14-27): 30-60 sec; OT: 2-3 min; SP: 2-3 min; CCK(8S): 1.5-2.5 min). For the NPP-caged peptides, responses were calculated within the time window in which peak responses to the corresponding parent peptide were obtained. Peak responses to photo-uncaging were calculated by taking the average current over 200 ms window surrounding the peak current measured for each cell. Time constants describing the photo-uncaging-evoked currents were obtained by fitting either a single exponential or double exponential function to the waveform of the average uncaging response for all cells in a given condition. Summary values are reported as mean ± SEM. Data were found to be normally distributed using a Shapiro-Wilk test and subsequently analyzed using an Ordinary one-way ANOVA followed by Sidak's test for multiple comparisons. All statistical tests were performed in GraphPad Prism v.9.5.1. Specific statistical tests and corrections are described for each figure in the text and figure legends.
